# From indoor psychotherapy and counseling to walk-and-talk: psychological and physiological responses across a fixed sequence of indoor, urban, and nature-based sessions

**DOI:** 10.3389/fpsyg.2026.1880259

**Published:** 2026-08-13

**Authors:** Katarína Matejová

**Affiliations:** Department of Psychology, Faculty of Arts and Letters, Catholic University in Ružomberok, Ružomberok, Slovakia

**Keywords:** environmental psychology, nature-based psychotherapy, psychophysiological response, therapeutic setting, walk-and-talk counseling, walk-and-talk therapy

## Abstract

**Background:**

Walk-and-talk psychotherapy and counseling have been proposed as meaningful alternatives or complements to conventional indoor practice, but quantitative evidence directly comparing short-term client responses across settings remains limited. This practice-based study examined whether immediate psychological and physiological responses varied across a fixed sequence of three psychotherapy/counseling sessions: indoor, urban walk-and-talk, and nature walk-and-talk.

**Methods:**

A within-subject repeated-measures design was used. Participants completed three measured psychotherapy/counseling sessions in a fixed order (indoor, urban, nature), with pre-and post-session assessments in each setting. The final analytical sample comprised 213 participants aged 14–74 years; analyses involving physical activity were based on 185 valid International Physical Activity Questionnaire cases. Repeated-measures analyses of variance tested preregistered differences in psychological and physiological change scores, and Pearson correlations examined associations with personality traits, environmental identity, and physical activity. Because setting was confounded with session order, findings are interpreted as setting-associated response patterns across a fixed sequence rather than as isolated causal effects of environmental context.

**Results:**

Short-term response patterns were differentiated rather than uniform. For psychological outcomes, Bonferroni-corrected effects were found for POMS Vigor, *F*(1.53, 324.68) = 53.50, *p* < 0.001, *η*^2^ = 0.146; POMS Dejection, *F*(1.89, 399.63) = 10.11, *p* < 0.001, *η*^2^ = 0.031; and PANAS Negative Affect, *F*(1.17, 246.99) = 8.55, *p* = 0.002, *η*^2^ = 0.020. The nature setting showed the largest increase in Vigor and the largest decrease in Dejection, whereas Negative Affect decreased least in the urban setting compared with indoor and nature settings. For physiological outcomes, Bonferroni-corrected effects were found for systolic blood pressure, *F*(1.35, 285.37) = 15.77, *p* < 0.001, *η*^2^ = 0.047; diastolic blood pressure, *F*(1.97, 417.53) = 10.74, *p* < 0.001, *η*^2^ = 0.031; and heart rate, *F*(1.91, 404.64) = 6.98, *p* = 0.001, *η*^2^ = 0.019. Blood pressure increased indoors but decreased slightly in both walk-and-talk psychotherapy/counseling settings. Preregistered individual-difference analyses yielded no robust Bonferroni-corrected associations.

**Conclusion:**

Immediate psychological and physiological responses varied across a fixed sequence of indoor, urban walk-and-talk, and nature walk-and-talk psychotherapy/counseling sessions. The findings do not support a simple conclusion that any one setting was globally superior. Rather, they suggest selective setting-associated response patterns, with nature walk-and-talk showing more favorable changes in selected short-term psychological outcomes and physiological responses following a different pattern. Because setting was confounded with session order, these findings should be interpreted cautiously.

## 1 Introduction

Walk-and-talk psychotherapy and counseling are more than therapy relocated outdoors; they represent modes of psychological intervention in which therapeutic and counseling processes unfold within a changing socio-physical environment. Walk-and-talk techniques in psychology involve delivering psychological intervention while walking outdoors (Cooley et al., 2021; Jordan and Marshall, 2010; Koziel et al., 2021; Shillito-Clarke, 2008). In the present study, we focus specifically on their use in psychological counseling and psychotherapy. Walk-and-talk counseling and psychotherapy represent a growing form of outdoor psychological practice in which therapist and client move together through an external environment, most often side by side rather than face to face (Cooley et al., 2021; Jordan, 2014; Revell and McLeod, 2017). Rather than constituting a distinct therapeutic school, this format is typically understood as pan-theoretical and potentially adaptable among different counseling and psychotherapeutic orientations (Jordan, 2014). It may serve either as the primary format of therapeutic work or as a complement to traditionally arranged indoor sessions (Berger, 2006; Berger and McLeod, 2006; Doucette, 2004; Jordan, 2014; Revell and McLeod, 2017). What makes this format scientifically important is that it brings the setting of therapy itself into view as a potentially active component of intervention.

From an environmental psychology perspective, the key issue is that walk-and-talk renders the socio-physical context of psychotherapy and counseling unusually visible. Conventional psychotherapy typically takes place indoors, where the physical setting is often treated as a relatively stable background to the therapeutic process. By contrast, walk-and-talk sessions unfold in dynamic outdoor environments that may vary in naturalness, sensory stimulation, privacy, social exposure, predictability, and perceived safety (Cooley et al., 2021; Jordan and Marshall, 2010; McLeod, 2013; Udler, 2023). Because of these variations, walk-and-talk psychotherapy and counseling provide a useful context for examining whether immediate psychological and physiological responses to psychotherapy/counseling differ depending on where the intervention takes place. In this sense, walk-and-talk is not only a clinical format but also a naturalistic test case for how therapeutic processes interact with environmental context.

A first reason to expect such differences comes from the literature on walking, movement, and psychological well-being. Previous research suggests that walking is associated with a range of potentially beneficial outcomes, including affective and cognitive restoration (Roe and Aspinall, 2010), improved working memory (Koselka et al., 2019), reduced depression and anxiety symptoms (Kotera et al., 2021; Robertson et al., 2012), improved mood (Koselka et al., 2019; Song et al., 2016), and support in coping with loss and loneliness (Muir and McGrath, 2018). Walking has also been discussed as a context that may support psychological processing (Hays, 1999), and, as a common and relatively accessible form of physical activity, it may facilitate flowing conversation without requiring specialized equipment or training (Janeczko et al., 2020; Koziel et al., 2021; Prince-Llewellyn and McCarthy, 2025; Raine et al., 2017; Revell et al., 2014). More broadly, walking has been described as a context that may support cerebral blood flow and psychological flexibility, a process often considered relevant to therapeutic change (Brandon et al., 2021; Carter et al., 2018). Taken together, this literature suggests that movement may shape psychotherapy and counseling not only behaviorally, but also affectively, cognitively, and physiologically.

A second reason to expect setting-related differences comes from research on the psychological and physiological effects of natural environments. A substantial number of studies suggest that spending time in nature may support restoration, improve mood, reduce stress, and promote well-being in both general and clinical populations (Berman et al., 2008; Bratman et al., 2015; Chalquist, 2009; Greenleaf et al., 2023; Jordan, 2014; Korpela et al., 2016; Lee et al., 2014; Milton, 2009; Morita et al., 2007; Raman et al., 2021; Stigsdotter et al., 2011). Studies comparing walking in different environments have also reported differences in physiological and psychological responses between urban and natural settings (Greene et al., 2017; Hassan et al., 2018; Janeczko et al., 2020; Lee et al., 2014; Roe and Aspinall, 2010; Song et al., 2016). This literature does not by itself establish the effectiveness of walk-and-talk psychotherapy or counseling, but it does suggest that both movement and environmental context may plausibly shape the immediate experience and short-term outcomes of psychotherapeutic and counseling work. The implication is not that nature is necessarily better, but that different environments may systematically afford different forms of immediate therapeutic response.

Walk-and-talk psychotherapy and counseling may therefore be understood as more than simply relocating “ordinary” therapy or counseling outdoors. Some authors have argued that when therapy moves from the consulting room to an outdoor setting, the therapeutic frame itself changes (Cooley et al., 2021; Jordan, 2014; Jordan and Marshall, 2010; McLeod, 2013). The therapist no longer fully controls the physical environment, the session unfolds in a public or semi-public space, and the side-by-side format may alter patterns of attention, disclosure, and interpersonal positioning (Jordan and Marshall, 2010; Langs, 2019; McLeod, 2013). The ethical framework may also require reconsideration, particularly regarding confidentiality, safety, professional boundaries, and the management of unpredictable events in outdoor environments (Hooley, 2016; Reese, 2016; Zur, 2001). At the same time, the changing frame may be experienced as beneficial, for example, by reducing formality, redistributing power, and facilitating a different quality of relational contact. Accordingly, the question is not only whether psychotherapy and counseling can be conducted outdoors, but also how outdoor settings may transform therapeutic and counseling processes.

Existing empirical work on walk-and-talk psychotherapy and counseling is promising, but the evidence base remains limited. Qualitative and mixed-method studies suggest that both clients and practitioners often experience outdoor talking therapy as meaningful, acceptable, and at times enriching (Cooley et al., 2021; Doucette, 2004; Greenleaf et al., 2023; Jordan and Marshall, 2010; Koziel et al., 2021; Newman and Gabriel, 2023; Prince-Llewellyn and McCarthy, 2025; Revell and McLeod, 2017; Shillito-Clarke, 2008; Udler, 2023). Revell and McLeod (2017) found that practitioners were often drawn to walk-and-talk because of personal belief in the value of walking and the outdoors, as well as a wish to broaden the methods available in therapy. At the same time, these studies suggest that outdoor therapy is not uniformly beneficial in all circumstances. Experiences may depend on the specific environmental context, the degree of privacy, the relational dynamics of the session, and the fit between setting and therapeutic task (Greenleaf et al., 2023; Newman and Gabriel, 2023). Thus, the emerging literature supports the relevance of outdoor psychotherapy and counseling while simultaneously indicating that their effects are likely to be conditional rather than universal.

What is still missing is direct quantitative evidence comparing psychotherapy and counseling responses across multiple settings within the same design. Much of the literature focuses either on walking and nature more generally or on qualitative reports of therapist and client experiences, rather than on direct comparison of psychotherapeutic and counseling responses across different settings using the same participants (Prince-Llewellyn and McCarthy, 2025; Revell and McLeod, 2017). Although there are partial studies examining walking in urban versus natural environments, and partial studies exploring experiences of walk-and-talk practice, there is still insufficient evidence comparing the same broad form of psychological intervention delivered across different settings—particularly indoor, urban outdoor, and natural outdoor contexts – using the same participants and a repeated-measures design (Cooley et al., 2021; Greenleaf et al., 2023; Janeczko et al., 2020; Lee et al., 2014; Newman and Gabriel, 2023; Roe and Aspinall, 2010; Song et al., 2016). Without such comparisons, it remains difficult to determine whether psychotherapeutic and counseling settings are associated with different immediate response patterns, and if so, in what ways.

This gap is important not only for evaluating the viability of walk-and-talk formats but also for clarifying how environmental context may shape intervention-related change. If the socio-physical setting of psychotherapy and counseling is not neutral, then differences across indoor, urban, and nature contexts may be expected not only in subjective affective responses, but also in short-term physiological regulation. At the same time, such effects may be selective rather than global. Different settings may support different aspects of immediate psychological and physiological response, rather than one setting emerging as uniformly superior across all outcomes. It is likewise possible that not all clients respond in the same way. Theoretical and practical discussions of walk-and-talk often imply that some individuals may benefit more than others, depending on personal characteristics, yet empirical evidence on these moderators remains sparse. In particular, it remains unclear whether individual differences such as personality traits, environmental identity, or physical activity are related to the magnitude of change associated with psychotherapy/counseling across settings. The central unresolved question, then, is not simply whether setting was associated with distinct response patterns, but how, for whom, and for which immediate results it matters.

The present study was designed to address these gaps. Specifically, we compared immediate pre-post psychological and physiological changes associated with psychological intervention across three settings: (1) a traditional indoor setting, (2) walk-and-talk in an urban environment, and (3) walk-and-talk in a natural environment. In addition, we examined whether personality traits, environmental identity, and physical activity were related to the magnitude of change across these settings. By doing so, the study aimed to contribute to the environmental psychology literature by testing whether the socio-physical setting of psychotherapy and counseling is associated with systematically different short-term psychological and physiological responses, and whether these responses differ as a function of selected individual characteristics. In this way, the study considers both the environmental question of whether the setting was associated with distinct response patterns and the clinical question of whether its relevance is likely to be selective rather than uniform.

For clarity and terminological consistency, the preregistered hypotheses are summarized below without changing their substantive meaning.

We hypothesized that immediate intervention-related change would differ across the three settings. First, we expected that psychological change would vary by setting, both for positive (H1a) and negative states (H1b). Second, we expected that physiological change would vary by setting, specifically for systolic blood pressure (H2a), diastolic blood pressure (H2b), and heart rate (H2c). Finally, we expected that the magnitude of setting-specific change would be related to selected individual-difference variables, namely personality traits (H3), environmental identity (H4), and physical activity (H5).

## 2 Methods

### 2.1 Design

The study used a practice-based within-subject repeated-measures design to examine immediate psychological and physiological responses across three psychotherapy/counseling sessions conducted in different socio-physical contexts: (1) a traditional indoor setting, (2) an urban walk-and-talk setting, and (3) a nature walk-and-talk setting. Each participant contributed data from all three settings, with psychological and physiological variables assessed immediately before and after each measured session.

The three settings were completed in a fixed sequence: indoor, urban walk-and-talk, and nature walk-and-talk. The design therefore allowed within-person comparison of short-term pre-post response patterns across the three measured sessions, but it did not allow the effects of setting to be separated from session order. Observed differences between settings may therefore reflect environmental context, therapeutic progression, adaptation to the study procedures, carryover effects, or other time-related processes. For this reason, the study is best understood as examining setting-associated response patterns across a fixed sequence of psychotherapy/counseling sessions rather than isolating causal effects of environmental setting alone.

For each setting, change scores were calculated as post-intervention minus pre-intervention values. Positive values therefore indicate an increase from pre-to post-session assessment, whereas negative values indicate a decrease. This change-score framework was used consistently across psychological and physiological outcomes.

### 2.2 Participants and recruitment

The study was conducted as a practice-based repeated-measures study involving psychologists and clients across routine counseling and psychotherapeutic care settings in Slovakia. Psychologists were recruited in two rounds through professional and statutory organizations in Slovakia, with the first recruitment round conducted in April 2025 and the second in August 2025. A total of 539 psychologists were contacted, and 24 psychologists agreed to participate in this pilot study. Based on available information, the participating professionals were predominantly counseling psychologists, clinical psychologists, and psychotherapists. Regarding workplace setting, 12 psychologists worked in counseling services (10 public, 2 private), 10 worked in outpatient practice (4 public, 6 private), and 2 worked in child care residential settings (Centres for Children and Families, CDR). Together, these settings represented both counseling and psychotherapeutic practice contexts. Throughout the manuscript, the term psychotherapy/counseling is used to refer to the broad psychological intervention context represented by the participating psychologists’ routine professional practice. Accordingly, the study was embedded in a heterogeneous but ecologically valid range of real-world service contexts.

Clients were recruited from ongoing routine practice rather than through a separate research-specific sampling frame. Recruitment was carried out by the participating psychologists among individuals already in their care. Participation was voluntary, and psychologists approached clients whom they considered potentially suitable for taking part in all three measured sessions, including the two walk-and-talk conditions. According to the available information, most psychologists approached all current clients whom they considered suitable. The target age range was 12 to 75 years. No centrally imposed diagnostic or symptom-based exclusion criteria were applied. The main practical requirement was that participants be able to complete an approximately 1-h walk. Suitability for participation was therefore determined by the treating psychologist on a case-by-case basis, taking into account the client’s health status, mobility, current clinical condition, and the appropriateness of walk-and-talk work within ongoing care.

This recruitment strategy was chosen to preserve the practice-based character of the study, but it also limits the representativeness of the sample. The final sample should therefore be understood as comprising clients who were already engaged in routine psychological care, were considered suitable for walk-and-talk participation by their psychologist, agreed to take part, and completed the full repeated-measures protocol. It does not represent all clients receiving psychotherapy or counseling, nor all clients for whom outdoor or walking-based formats may or may not be clinically appropriate.

**Figure 1:**
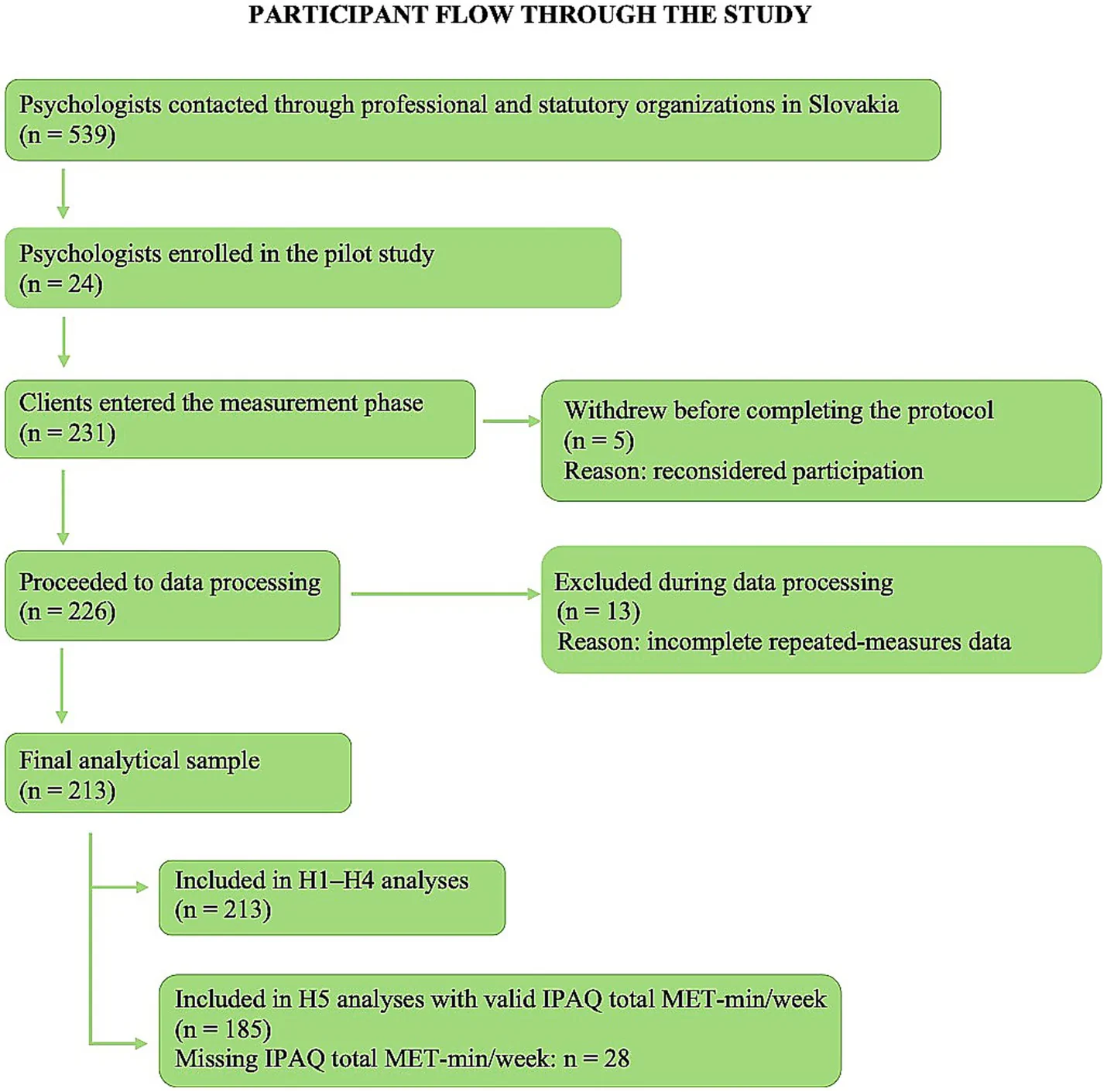
Participant flow through the study. The diagram presents the progression from psychologist recruitment to the final analytical sample. Clients were recruited by participating psychologists from among individuals already in their care. Five participants withdrew before completing the protocol; according to the participating psychologists, these withdrawals occurred because participants reconsidered participation or found the measurement procedures uncomfortable. An additional 13 cases were excluded during data processing because the repeated-measures dataset was incomplete, for example due to missing pre-or post-session psychological state data in one of the settings and/or missing physiological measurements. The final analytical sample for repeated-measures analyses comprised 213 participants. Analyses involving total physical activity were based on 185 participants with valid IPAQ total MET-min/week data.

The final analytical sample was defined by completion of the repeated-measures protocol and data completeness. The final analytical sample comprised 213 participants who completed all three measured sessions. Four additional participants who had initially entered the study withdrew before completing the protocol because they reconsidered participation or found the measurement procedures uncomfortable. In addition, 13 cases (5.8%) were excluded during data processing because they did not contain complete repeated-measures data for all variables required for analysis, such as missing pre-or post-session psychological state data in one of the settings and/or missing physiological measurements. The total number of clients originally approached was not systematically recorded. Participants were recruited and assessed within the routine practice of 24 psychologists, such that individual participants were partially nested within treating psychologists. The resulting sample therefore reflects both the strengths and the constraints of research conducted within routine practice conditions. The flow of psychologist recruitment, participant inclusion, withdrawals, exclusions, and final analytical samples is shown in Figure **1**.

### 2.3 Procedure

Data collection was organized to ensure that the study protocol was implemented as consistently as possible across participating psychologists. Data collection took place between May and November 2025. Before the start of data collection, the first author met individually with each participating psychologist and provided detailed training in the study protocol, including the order of settings, the order of measurements, and the administration of study materials. Each psychologist received the informed consent forms and study instructions. For participants under 18 years of age, informed consent was also obtained from a parent or legal guardian. This preparation was intended to maximize procedural consistency across sites and practitioners.

The study was embedded in routine counseling or psychotherapy rather than constructed as a manualized intervention trial. All measured sessions took place with the psychologist whom the client was already attending in regular practice. The content of sessions was not standardized by the research protocol and reflected the psychologist’s usual professional practice. The study therefore assessed the same broad format of psychological intervention across settings rather than a manualized intervention with fixed content. Thus, the study compared intervention delivery across settings under routine practice conditions rather than testing a manualized intervention with identical content across therapists. The aim was therefore to capture setting-related differences under ecologically valid counseling and psychotherapeutic conditions rather than under experimentally standardized therapeutic content.

Baseline individual-difference measures were collected before the repeated session-based assessments began. Before the first measured session, participants completed the NEO-FFI, Environmental Identity, and IPAQ questionnaires using a standardized administration procedure. These questionnaires were completed electronically on a mobile phone and were completed independently by the clients themselves. To preserve anonymity, each participant created a self-generated code; this code was used to link measurements across sessions, but it was not written on the informed consent form. This procedure allowed repeated measurements to be linked while preserving participant anonymity.

The core study procedure consisted of three repeatedly measured psychotherapy/counseling sessions conducted in a fixed order. The measured sessions were conducted in the following sequence: first, the indoor setting; then, the urban walk-and-talk setting; and finally, the nature walk-and-talk setting. This order was selected before data collection to maintain a consistent procedure across participating psychologists and to introduce the outdoor formats gradually, beginning with the most familiar and routinely used therapeutic context. In the practice-based context of the study, counterbalancing was not implemented because the study was embedded in ongoing care across multiple service settings rather than being organised as a laboratory-based or fully controlled intervention trial. A fixed sequence was therefore used to reduce procedural heterogeneity and to support feasible implementation by participating psychologists.

This design selection has important implications for interpretation. Because all participants completed the settings in the same order, setting is confounded with session order. Differences among the three measured sessions, therefore, cannot be exclusively attributed to the socio-physical environment. They may also reflect therapeutic progression, increasing familiarity with the measurement procedure, adaptation to the study protocol, expectancy effects, carryover between sessions, or changes in the client–psychologist relationship over time. Accordingly, the study should be interpreted as comparing immediate response patterns across a fixed sequence of indoor, urban walk-and-talk, and nature walk-and-talk sessions, rather than as isolating causal effects of environmental setting alone.

Repeated state and physiological assessments were obtained immediately before and after each measured session. Blood pressure and heart rate were assessed before and after the session, and participants completed electronic questionnaires measuring current psychological state at both time points. Sessions usually occurred at approximately 1-to 2-week intervals, although longer intervals were sometimes necessary due to routine scheduling constraints. This procedure made it possible to examine short-term pre-post change within each psychotherapy/counseling setting while preserving the rhythm and practical conditions of ongoing care.

### 2.4 Setting characteristics

The study compared three broad counseling and psychotherapeutic settings: an indoor setting, an urban walk-and-talk setting, and a nature walk-and-talk setting. The indoor setting reflected the usual practice conditions of each participating psychologist and therefore varied according to the facilities available at the respective workplace, such as a counseling room, outpatient office, or other routinely used therapeutic space.

The urban walk-and-talk setting was defined as a walking session conducted in a built environment, typically within a town or city. The nature walk-and-talk setting was defined as a walking session conducted in a predominantly natural environment, most often outside the town/city or in a large park where buildings were not visibly dominant. These categories were intended to capture ecologically meaningful practice settings rather than experimentally standardized environmental exposures.

Routes were selected by the participating psychologists before data collection. To increase within-practitioner consistency, each psychologist used the same urban route and the same nature-based route with all participating clients whenever feasible. However, routes necessarily differed between psychologists because data collection took place across different practice locations. The study therefore standardized route use within practitioners but did not impose identical routes across practitioners.

Walk-and-talk sessions lasted approximately 50–60 min, corresponding to the duration of the measured indoor sessions. Outdoor sessions were conducted under routine practice conditions. Weather conditions and potential disturbing factors during outdoor sessions were recorded as contextual information. However, these variables were not included as preregistered predictors in the primary analyses. Other environmental characteristics, such as noise level, density of passers-by, privacy, sensory load, perceived safety, route predictability, and perceived restorativeness, were not systematically quantified.

Accordingly, the urban and nature conditions should be interpreted as broad socio-physical practice contexts rather than tightly controlled environmental manipulations. Differences between outdoor settings may have reflected not only the degree of naturalness, but also other co-occurring environmental features, including privacy, stimulation, social exposure, route characteristics, and weather. This limitation is particularly relevant for physiological outcomes, which may be sensitive to environmental conditions as well as to walking, emotional engagement, and the therapeutic interaction itself.

### 2.5 Measures

#### 2.5.1 Psychological state measures

Immediate psychological response to psychotherapy/counseling was assessed using repeated state measures administered in every setting. Psychological outcomes were assessed separately for each setting and time point using the following state measures. In all cases, participants responded to the instruction “How do you feel right now?”

Positive and Negative Affect Schedule (PANAS; Watson et al., 1988), yielding Positive Affect and Negative Affect scores, each computed as the mean of 10 items. Items were rated on a 1–5 scale, with higher scores indicating greater current positive and Negative Affect, respectively.

Profile of Mood States, 16-item version (POMS-16; Petrowski et al., 2021; based on McNair et al., 1971), yielding four subscales: Vigor, Anger, Fatigue, and Dejection, each computed as the mean of four items. Items were rated on a 0–4 scale, with higher scores indicating greater intensity of the respective mood state.

Zuckerman Inventory of Personal Reactions (ZIPERS; Zuckerman, 1977), yielding five dimensions: Positive Affect, Attention, Fear, Sadness, and Anger. Positive Affect was calculated as the mean of four items, Attention as the mean of two items including one reverse-coded item, Fear as the mean of three items, Sadness as a single-item indicator, and Anger as the mean of two items. Items were rated on a 1–5 scale, with higher scores indicating greater intensity of the respective state.

State–Trait Inventory for Cognitive and Somatic Anxiety (STICSA; Ree et al., 2008), yielding Cognitive Anxiety and Somatic Anxiety, computed as the mean of 11 and 10 items, respectively. Items were rated on a 1–4 scale, with higher scores indicating greater current anxiety in the respective domain.

All state measures were administered before and after each session in each setting. Reverse-coded items were transformed before score aggregation. Unless otherwise stated, all scale scores were computed as mean scores. Pre-intervention, post-intervention, and change scores were derived for each setting. Psychometric characteristics of the author-translated versions used in the present study are reported in the Supplementary material 1 (Supplementary Tables S1–S4). Together, these measures were intended to capture multiple facets of short-term emotional and psychological state change across psychotherapeutic and counseling contexts. Because Slovak validated versions were not available for all state instruments used in the repeated session assessments, author-translated Slovak versions were used. These translations were prepared to preserve the content of the original items and to allow repeated administration under routine practice conditions; however, formal validation of these translated state measures was beyond the scope of the present study. Internal consistency estimates for the translated administrations are therefore reported in Supplementary material 1 and are considered when interpreting the findings. This is particularly relevant for scales and administrations showing reduced reliability, where associations may have been attenuated and non-significant findings should be interpreted cautiously.

#### 2.5.2 Physiological measures

Physiological response to psychotherapy/counseling was assessed through repeated blood pressure and heart rate measurements in each setting. Blood pressure and heart rate were measured using the Huawei Watch D. For blood pressure measurement, the Huawei Watch D has been validated for adult blood pressure assessment according to the ANSI/AAMI/ISO 81060-2:2018 standard and is listed as a validated home blood pressure monitor in the STRIDE BP database. Accordingly, blood pressure readings were obtained using a validated device. The blood pressure device has been validated for adult blood pressure assessment; therefore, readings from adolescent participants should be interpreted with additional caution. All psychologists involved in data collection were trained by the first author before the start of the study and used the same standardized measurement protocol throughout the study.

Physiological outcomes included systolic blood pressure, diastolic blood pressure, and heart rate measured before and after the intervention in each setting. Physiological measurements were obtained according to a standardized protocol with participants in a seated position after 5 min of rest. For outdoor sessions, measurements were taken outdoors using the same procedure before and after each session. Heart rate was measured twice at each assessment point, and the session value was calculated as the mean of the two readings. For blood pressure, two readings were obtained routinely at each time point. A third reading was taken only when the discrepancy between the first and second measurements exceeded 10 mmHg for systolic blood pressure or 6 mmHg for diastolic blood pressure. Session values for systolic and diastolic blood pressure were then calculated as the mean of the available readings. For each physiological variable, change scores were computed as post-intervention minus pre-intervention values. Physiological outcomes are reported in the Supplementary material 1 (Supplementary Table S5). These measures allowed the study to capture not only subjective but also short-term bodily correlates of psychotherapy/counseling across settings. Because the outdoor sessions involved walking and took place in naturally varying environments, the physiological measures should be understood as indicators of short-term post-session bodily state within each psychotherapy/counseling context. They do not isolate the independent physiological effects of environmental exposure, walking, or therapeutic interaction.

#### 2.5.3 Personality traits

Personality traits were assessed as relatively stable individual-difference variables that might shape responses to psychotherapeutic and counseling settings. Personality traits were assessed using the NEO Five-Factor Inventory (NEO-FFI; Costa and McCrae, 1992) in the Slovak translation by Ruisel and Halama (2007). The instrument yields five domain scores: Neuroticism, Extraversion, Openness to Experience, Agreeableness, and Conscientiousness. Each domain consisted of 12 items, rated on a 0–4 scale. Final domain scores were computed as the mean of the respective items after reverse-coded items had been transformed. Higher scores indicate a higher level of the respective personality trait. The instrument was administered once before the measured sessions. Psychometric characteristics of the version used in the present study are reported in the Supplementary material 1 (Supplementary Table S6). In this way, personality was treated as a potential moderator of setting-specific change rather than as an outcome of intervention.

#### 2.5.4 Environmental identity

Environmental identity was included as an individual-difference construct directly relevant to the environmental focus of the study. Environmental identity was assessed using the revised Environmental Identity Scale (Clayton et al., 2021). The version used in the present study comprised 14 items, rated on a 1–7 scale. A Slovak version was prepared using back-translation, and the total score was computed as the mean of all 14 items, with higher scores indicating stronger environmental identity. The instrument was administered once before the measured sessions. Psychometric characteristics of the translated version used in the present study are reported in the Supplementary material 1 (Supplementary Table S7). This measure was intended to test whether a person’s identification with nature-related experience was associated with setting-specific psychotherapy/counseling response.

#### 2.5.5 Physical activity

Physical activity was assessed as both a continuous behavioral characteristic and an exploratory categorical grouping variable. Physical activity was assessed using the International Physical Activity Questionnaire, Short Form (IPAQ-SF; Craig et al., 2003), referring to physical activity during the previous 7 days. Scoring followed the IPAQ Research Committee guidelines, with total physical activity expressed as MET-min/week and activity levels classified as low, moderate, or high (IPAQ Research Committee, 2005). The questionnaire was administered as a self-report measure, either electronically or in paper format. Derived variables represented weekly frequency (days/week) and daily duration (minutes/day) of vigorous activity, moderate activity, and walking. Data preprocessing followed a missing-data approach, meaning that invalid or non-informative responses were treated as missing rather than recoded as zero. Specifically, days were restricted to the range 0–7, values below 10 min/day were treated as missing, values above 180 min/day were truncated to 180, and non-informative responses (e.g., “I do not know”) were treated as missing. Total physical activity was expressed as MET-min/week using standard IPAQ coefficients (8.0 for vigorous activity, 4.0 for moderate activity, and 3.3 for walking). In addition, participants were classified into low (<600 MET-min/week), moderate (600–2,999 MET-min/week), and high (≥3,000 MET-min/week) activity levels according to standard IPAQ scoring rules. In the confirmatory analyses, physical activity was operationalized continuously as IPAQ total MET-min/week; the categorical IPAQ activity level variable was used only in exploratory follow-up analyses. This dual operationalization allowed physical activity to be examined both as a graded individual-difference variable and as an exploratory activity-level grouping factor.

Response formats, scoring details, and psychometric characteristics of these measures are reported in the Supplementary material 1 (Supplementary Tables S8, S9).

### 2.6 Statistical analysis

The study hypotheses and primary analytical strategy were preregistered before data analysis on the Open Science Framework. For peer review, the preregistration is available through an anonymized view-only OSF link: https://osf.io/8v3w5/overview?view_only=61f53194f15943958f361870eeae742b. The analytic strategy was designed to follow the preregistration while remaining transparent about the practical choices required by the available software environment. Analyses were conducted in jamovi (version 2.7.27; RRID: SCR_016142) rather than in SPSS version 26, as originally stated in the preregistration; this change concerned the software platform only and did not alter the preregistered analytical approach.

Before score aggregation, reverse-coded items were recoded in the appropriate direction. Unless otherwise stated, scale scores were computed as mean scores of their constituent items. For repeated pre-and post-intervention assessments, all change scores were calculated uniformly as post-intervention minus pre-intervention values. Non-rounded values were retained where necessary to preserve analytical precision. All subsequent analyses were therefore based on a uniform and explicitly defined change-score framework.

The primary confirmatory analyses tested whether pre-post change differed across the three measured sessions, which were conducted in a fixed sequence corresponding to the indoor, urban walk-and-talk, and nature walk-and-talk settings. Repeated-measures analyses of variance (ANOVAs) were conducted separately for each preregistered outcome, with setting specified as a three-level within-subject factor. Because the setting was confounded with session order, statistically significant setting-related differences should be interpreted as differences within this fixed sequence of psychotherapy/counseling sessions rather than as isolated causal effects of environmental setting. Mauchly’s test was used to evaluate sphericity. When the assumption of sphericity was violated, Greenhouse–Geisser-corrected degrees of freedom were reported. For significant omnibus effects, Bonferroni-corrected pairwise comparisons were examined to identify which settings differed.

In line with the preregistration, Bonferroni correction was additionally applied across the 16 preregistered omnibus tests (13 psychological outcomes and 3 physiological outcomes). Adjusted omnibus *p* values were calculated as pBonf = min (p × 16, 1.00), which is equivalent to an adjusted alpha level of 0.003125. Effect sizes for the repeated-measures ANOVAs were reported as eta squared (*η*^2^), consistent with the preregistration. This approach allowed the central setting hypotheses to be tested conservatively across a broad outcome set.

The repeated-measures ANOVA framework followed the preregistered primary analytic plan and was retained as the main inferential approach. This framework models within-person differences across the three measured sessions, but it does not explicitly account for the partially nested structure of the data, in which participants were recruited and treated by 24 psychologists. Because the analytic dataset did not include a reliably usable therapist identifier, therapist-level variance was not modeled statistically. The possible contribution of therapist-level factors, including therapeutic orientation, interpersonal style, route selection, and implementation differences, is therefore considered when interpreting the findings.

The confirmatory moderator analyses focused on whether individual-difference variables were associated with setting-specific change. To test the preregistered individual-difference hypotheses (H3, H4, and H5), Pearson correlation analyses were conducted between the relevant predictor variables and all preregistered setting-specific change scores. For H3, the five NEO-FFI domains were correlated with all setting-specific change scores, yielding 240 preregistered tests (5 traits x 48 change scores). For H4, Environmental Identity was correlated with the same 48 setting-specific change scores, yielding 48 tests. For H5, total physical activity, operationalized as IPAQ total MET-min/week, was correlated with the same 48 setting-specific change scores, again yielding 48 tests. Bonferroni-adjusted *p*-values were calculated within each hypothesis as a conservative control of family-wise error [H3: pBonf = min (p × 240, 1.00); H4/H5: pBonf = min (p × 48, 1.00)]. In the confirmatory H5 analyses, physical activity was operationalized continuously as IPAQ total MET-min/week; the categorical IPAQ activity level analysis was conducted only as an exploratory follow-up. Thus, the confirmatory individual-difference tests were designed to be broad in coverage but stringent in inference.

The Bonferroni correction was chosen in line with the preregistered analytic strategy to provide conservative control of family-wise error across a broad set of outcomes and individual-difference tests. This approach lowers the risk of false-positive findings, which was particularly important given the large number of preregistered comparisons. At the same time, it increases the risk of a Type II error, especially in individual-difference analyses, where many small associations were tested. Accordingly, non-significant Bonferroni-corrected moderator findings should be interpreted as an lack of robust evidence under a stringent correction procedure, rather than as clear evidence that the examined individual-difference variables are unrelated to setting-specific change.

Exploratory analyses were used to examine whether categorical physical activity level showed additional associations with setting-related change. As an exploratory follow-up to the preregistered H5 analyses, mixed ANOVAs were additionally conducted with setting as a within-subject factor and IPAQ activity level (low, moderate, high) as a between-subject factor. These analyses were not part of the preregistered primary testing strategy and are therefore reported separately from the confirmatory analyses. Effect sizes for these exploratory mixed ANOVAs were reported as partial eta squared (ηp^2^), following the software output. These analyses were intended to extend, rather than replace, the preregistered continuous operationalization of physical activity.

Supplementary paired TOST analyses were conducted for the three planned setting contrasts to examine equivalence or minimum effects in the original outcome metrics. Because omnibus equivalence testing was not available in the software environment, these analyses were implemented at the contrast level. Outcome-specific equivalence bounds, and full results are reported in Supplementary material 2 (Supplementary Table S14).

## 3 Results

### 3.1 Sample characteristics

The final analytical sample consisted of 213 participants aged 14 to 74 years (M = 29.64, SD = 12.99). The preregistered eligibility criteria allowed participants aged 12–75 years. However, no participants younger than 14 years were included in the final analytical sample, resulting in an observed sample age range of 14–74 years. The sample included 59 men (27.7%), 150 women (70.4%), and 4 participants (1.9%) who preferred not to disclose their gender. Complete psychological and physiological change scores were available for all participants. Analyses involving physical activity were based on 185 valid IPAQ cases because 28 participants had missing IPAQ total MET values. In addition to four withdrawals before completion of the protocol, 13 further cases were excluded during data processing because of incomplete repeated-measures data. Thus, all primary repeated-measures analyses were based on the same final sample, except for analyses involving IPAQ-derived physical activity.

#### 3.1.1 Note on scoring and psychometric characteristics

Unless otherwise stated, all change scores were calculated as post-intervention minus pre-intervention values, such that positive values indicate an increase and negative values indicate a decrease from pre-to post-intervention. All scale scores were computed as mean scores of their constituent items, and reverse-coded items were transformed prior to aggregation. Detailed descriptive and psychometric characteristics of all measures are reported in the Supplementary material (Supplementary material 1). Because several state measures were administered using author-translated Slovak versions and selected administrations showed reduced internal consistency, findings involving these scales should be interpreted with caution. This applies particularly to PANAS Positive Affect in the urban setting, selected POMS administrations, several ZIPERS subscales, and Environmental Identity. Low or unstable reliability may have attenuated associations and may partly contribute to non-significant findings, especially in the individual-difference analyses. Supplementary paired TOST analyses for the three planned setting contrasts are reported in Supplementary material 2 (Supplementary Table S14).

### 3.2 Psychological state changes across settings

Repeated-measures ANOVAs were conducted to test whether psychological change scores differed across the three measured sessions corresponding to the indoor, urban walk-and-talk, and nature walk-and-talk settings. Greenhouse–Geisser-corrected degrees of freedom and *p* values are reported where applicable. In line with the preregistration, Bonferroni correction was applied across the 16 preregistered omnibus tests. Effect sizes are reported as eta squared (*η*^2^), consistent with the preregistration. The repeated-measures ANOVA results for all preregistered psychological outcomes are presented in Table 1.

**Table 1:** Repeated-measures ANOVA results for preregistered psychological outcomes across indoor, urban walk-and-talk, and nature walk-and-talk settings.

Hypothesis	Outcome	df1	df2	*F*	*p*	pBonf (omnibus)	*η* ^2^
H1a	Vigor (POMS)	1.53	324.68	53.50	< 0.001	**<0.001**	0.146
H1a	Positive affect (PANAS)	1.55	329.58	4.12	0.026	0.412	0.007
H1a	Positive affect (ZIPERS)	1.79	379.29	1.28	0.276	1.000	0.004
H1a	Attention (ZIPERS)	1.92	406.74	1.88	0.156	1.000	0.006
H1b	Negative affect (PANAS)	1.17	246.99	8.55	0.002	**0.038**	0.020
H1b	Dejection (POMS)	1.89	399.63	10.11	< 0.001	**0.001**	0.031
H1b	Anger (POMS)	1.89	400.33	0.92	0.396	1.000	0.003
H1b	Fatigue (POMS)	1.98	419.11	0.03	0.966	1.000	<0.001
H1b	Fear (ZIPERS)	1.54	327.24	0.41	0.608	1.000	0.001
H1b	Sadness (ZIPERS)	1.60	338.33	0.86	0.402	1.000	0.003
H1b	Anger (ZIPERS)	1.75	370.72	0.13	0.855	1.000	<0.001
H1b	Cognitive anxiety (STICSA)	1.96	414.69	0.29	0.741	1.000	0.001
H1b	Somatic anxiety (STICSA)	1.85	392.42	1.91	0.153	1.000	0.006

*N* = 213. Repeated-measures ANOVAs were conducted for preregistered psychological change scores across the three settings. Change scores were calculated as post-intervention minus pre-intervention values. Greenhouse–Geisser-corrected degrees of freedom are reported. Bonferroni-corrected *p* values reflect correction across the 16 preregistered omnibus tests. POMS, profile of mood states; PANAS, positive and negative affect schedule; ZIPERS, Zuckerman Inventory of Personal Reactions; STICSA, State–Trait Inventory for Cognitive and Somatic Anxiety; *η*^2^, eta squared; pBonf, Bonferroni-corrected *p*-value. Effect sizes are reported as eta squared (*η*^2^), in line with the preregistration. After Bonferroni correction, significant setting-related differences remained for vigor (POMS), negative affect (PANAS), and dejection (POMS). Bold values indicate omnibus effects that remained statistically significant after Bonferroni correction across the 16 preregistered omnibus tests.

#### 3.2.1 Positive psychological states

For positive psychological outcomes, only POMS Vigor showed a statistically significant setting-related difference after Bonferroni correction, *F*(1.53, 324.68) = 53.50, *p* < 0.001, *η*^2^ = 0.146, pBonf < 0.001. Mean change scores were 0.16 in the indoor setting, 0.26 in the urban walk-and-talk setting, and 1.05 in the nature walk-and-talk setting. Bonferroni-corrected pairwise comparisons showed that the nature setting differed from both the indoor (pBonf < 0.001) and urban (pBonf < 0.001) settings, whereas the indoor and urban settings did not differ (pBonf = 0.367).

PANAS Positive Affect showed a small uncorrected effect of setting, *F*(1.55, 329.58) = 4.12, *p* = 0.026, *η*^2^ = 0.007, with mean change scores of 0.23 (indoor), 0.35 (urban), and 0.26 (nature), but this effect did not survive Bonferroni correction (pBonf = 0.412). No significant setting-related differences were found for ZIPERS Positive Affect, *F*(1.79, 379.29) = 1.28, *p* = 0.276, *η*^2^ = 0.004, pBonf = 1.000, or ZIPERS Attention, *F*(1.92, 406.74) = 1.88, *p* = 0.156, *η*^2^ = 0.006, pBonf = 1.000.

Taken together, H1a was partially supported. Robust Bonferroni-corrected differences across the three measured sessions were observed only for POMS Vigor, with the largest increase observed in the nature walk-and-talk session.

#### 3.2.2 Negative psychological states

For negative psychological outcomes, significant setting-related differences that survived Bonferroni correction were observed for PANAS Negative Affect and POMS Dejection.

For PANAS Negative Affect, the effect of setting was significant, *F*(1.17, 246.99) = 8.55, *p* = 0.002, *η*^2^ = 0.020, pBonf = 0.038. Mean change scores were-0.32 in the indoor setting, −0.14 in the urban walk-and-talk setting, and-0.32 in the nature walk-and-talk setting. Bonferroni-corrected pairwise comparisons showed significant differences between the indoor and urban settings (pBonf = 0.009) and between the urban and nature settings (pBonf = 0.009), whereas the indoor and nature settings did not differ (pBonf = 1.000).

For POMS Dejection, the effect of setting was also significant, *F*(1.89, 399.63) = 10.11, *p* < 0.001, *η*^2^ = 0.031, pBonf = 0.001. Mean change scores were −0.29 in the indoor setting, −0.29 in the urban walk-and-talk setting, and −0.52 in the nature walk-and-talk setting. Bonferroni-corrected pairwise comparisons indicated that the nature setting differed from both the indoor setting (pBonf = 0.001) and the urban setting (pBonf = 0.001), whereas the indoor and urban settings did not differ (pBonf = 1.000).

No significant setting-related differences were found for POMS Anger, *F*(1.89, 400.33) = 0.92, *p* = 0.396, *η*^2^ = 0.003, pBonf = 1.000; POMS Fatigue, *F*(1.98, 419.11) = 0.03, *p* = 0.966, *η*^2^ < 0.001, pBonf = 1.000; ZIPERS Fear, *F*(1.54, 327.24) = 0.41, *p* = 0.608, *η*^2^ = 0.001, pBonf = 1.000; ZIPERS Sadness, *F*(1.60, 338.33) = 0.86, *p* = 0.402, *η*^2^ = 0.003, pBonf = 1.000; ZIPERS Anger, *F*(1.75, 370.72) = 0.13, *p* = 0.855, *η*^2^ < 0.001, pBonf = 1.000; STICSA Cognitive Anxiety, *F*(1.96, 414.69) = 0.29, *p* = 0.741, *η*^2^ = 0.001, pBonf = 1.000; or STICSA Somatic Anxiety, *F*(1.85, 392.42) = 1.91, *p* = 0.153, *η*^2^ = 0.006, pBonf = 1.000.

Taken together, H1b was partially supported. Robust Bonferroni-corrected differences across the three measured sessions were detected for PANAS Negative Affect and POMS Dejection, but not for the remaining preregistered negative-state outcomes.

#### 3.2.3 Summary of psychological state findings

Overall, H1 was partially supported. The strongest Bonferroni-corrected evidence for differences across the three measured sessions was found for POMS Vigor, POMS Dejection, and PANAS Negative Affect. No robust setting-related differences were observed for the remaining psychological outcomes after Bonferroni correction. Descriptive statistics and Bonferroni-corrected pairwise comparisons for the psychological outcomes that remained significant after correction are presented in Table 2.

**Table 2:** Descriptive statistics and pairwise comparisons for psychological outcomes that remained significant after Bonferroni correction.

Outcome	Indoor M (SD)	Urban w-a-t M (SD)	Nature w-a-t M (SD)	Significant pairwise comparisons
Vigor (POMS)	0.16 (0.72)	0.26 (0.61)	1.05 (1.37)	Indoor vs. nature, *p* < 0.001; urban vs. nature, *p* < 0.001
Dejection (POMS)	−0.29 (0.50)	-0.29 (0.54)	−0.52 (0.74)	Indoor vs. nature, *p* = 0.001; urban vs. nature, *p* = 0.001
Negative affect (PANAS)	−0.32 (0.54)	−0.14 (0.73)	−0.32 (0.56)	Indoor vs. urban, *p* = 0.009; urban vs. nature, *p* = 0.009

*N* = 213. Values are change scores calculated as post-intervention minus pre-intervention values. Positive values indicate an increase from pre-to post-intervention, whereas negative values indicate a decrease. w-a-t, walk-and-talk; POMS, Profile of Mood States; PANAS, Positive and Negative Affect Schedule. Pairwise comparisons were Bonferroni-corrected within each outcome across the three setting contrasts. Non-significant pairwise comparisons were as follows: vigor, indoor versus urban walk-and-talk, *p* = 0.367; dejection, indoor versus urban walk-and-talk, *p* = 1.000; negative affect, indoor versus nature walk-and-talk, *p* = 1.000.

### 3.3 Physiological changes across settings

Repeated-measures ANOVAs were done to test whether physiological change scores differed across the three measured sessions corresponding to the indoor, urban walk-and-talk, and nature walk-and-talk settings. Greenhouse–Geisser-corrected degrees of freedom and *p*-values are reported where applicable, and Bonferroni correction was applied in line with the preregistration. Descriptive statistics, omnibus ANOVA results, and Bonferroni-corrected pairwise comparisons for the physiological outcomes are presented in Table 3.

**Table 3:** Descriptive statistics and pairwise comparisons for preregistered physiological outcomes across indoor, urban walk-and-talk, and nature walk-and-talk settings.

Outcome	Indoor M (SD)	Urban w-a-t M (SD)	Nature w-a-t M (SD)	df1	df2	*F*	*p*	pBonf (omnibus)	*η* ^2^	Significant pairwise comparisons
Systolic blood pressure	3.77 (13.53)	−0.95 (6.08)	−0.48 (7.64)	1.35	285.37	15.77	<0.001	<0.001	0.047	Indoor vs. urban, *p* < 0.001; indoor vs. nature, *p* = 0.001
Diastolic blood pressure	1.11 (5.17)	−1.12 (5.36)	−0.87 (6.13)	1.97	417.53	10.74	<0.001	0.001	0.031	Indoor vs. urban, *p* < 0.001; indoor vs. nature, *p* = 0.001
Heart rate	−3.49 (10.30)	−2.70 (6.25)	−0.70 (8.02)	1.91	404.64	6.98	0.001	0.020	0.019	Indoor vs. nature, *p* = 0.002; urban vs. nature, *p* = 0.011

*N* = 213. w-a-t, walk-and-talk; *η*^2^, eta squared; pBonf, Bonferroni-corrected p value. Values are change scores calculated as post-intervention minus pre-intervention values. Greenhouse–Geisser-corrected degrees of freedom are reported. Bonferroni-corrected omnibus *p* values reflect correction across the 16 preregistered omnibus tests. Effect sizes are reported as eta squared (*η*^2^), in line with the preregistration. Pairwise comparisons were Bonferroni-corrected within each outcome across the three setting contrasts. Non-significant comparisons were as follows: systolic blood pressure, urban vs nature, *p* = 1.000; diastolic blood pressure, urban vs nature, *p* = 1.000; heart rate, indoor vs urban, *p* = 0.997. Blood pressure session values were computed as the mean of the available two or three readings; a third reading was obtained only when the discrepancy between the first and second readings exceeded the predefined threshold. Heart rate values were computed as the mean of two readings.

#### 3.3.1 Systolic blood pressure

A significant setting-related difference was found for systolic blood pressure change, *F*(1.35, 285.37) = 15.77, *p* < 0.001, *η*^2^ = 0.047, pBonf < 0.001. Mean change scores were 3.77 in the indoor setting, −0.95 in the urban walk-and-talk setting, and −0.48 in the nature walk-and-talk setting. Bonferroni-corrected pairwise comparisons showed that the indoor setting differed from the urban setting (pBonf < 0.001) and from the nature setting (pBonf = 0.001), whereas the urban and nature settings did not differ (pBonf = 1.000). Thus, H2a was supported, indicating that systolic blood pressure change differed across the three measured sessions.

#### 3.3.2 Diastolic blood pressure

A significant setting-related difference was found for diastolic blood pressure change, *F*(1.97, 417.53) = 10.74, *p* < 0.001, *η*^2^ = 0.031, pBonf = 0.001. Mean change scores were 1.11 in the indoor setting, −1.12 in the urban walk-and-talk setting, and −0.87 in the nature walk-and-talk setting. Bonferroni-corrected pairwise comparisons showed that the indoor setting differed from the urban setting (pBonf < 0.001) and from the nature setting (pBonf = 0.001), whereas the urban and nature settings did not differ (pBonf = 1.000). Thus, H2b was supported, indicating that diastolic blood pressure change differed across the three measured sessions.

#### 3.3.3 Heart rate

A significant setting-related difference was found for heart rate change, *F*(1.91, 404.64) = 6.98, *p* = 0.001, *η*^2^ = 0.019, pBonf = 0.020. Mean change scores were-3.49 in the indoor setting, −2.70 in the urban walk-and-talk setting, and −0.70 in the nature walk-and-talk setting. Bonferroni-corrected pairwise comparisons showed that the nature setting differed from the indoor setting (pBonf = 0.002) and from the urban setting (pBonf = 0.011), whereas the indoor and urban settings did not differ (pBonf = 0.997). Thus, H2c was supported, indicating that heart rate change differed across the three measured sessions.

#### 3.3.4 Summary of physiological findings

Overall, H2 was supported. Physiological change scores differed across the three measured sessions for systolic and diastolic blood pressure and heart rate. These results indicate that short-term physiological responses varied across the fixed sequence of indoor, urban walk-and-talk, and nature walk-and-talk sessions.

### 3.4 Individual-difference correlates of setting-specific change

#### 3.4.1 Personality traits

To test H3, Pearson correlation analyses were conducted between the five NEO-FFI dimensions and all setting-specific preregistered change scores. Across the 240 tested associations, 11 correlations reached nominal significance at *p* < 0.05. However, none remained significant after Bonferroni correction. The strongest nominal association was observed between Openness to Experience and STICSA Somatic Anxiety change in the nature setting, *r* = −0.211, *p* = 0.0019, pBonf = 0.463. Nominally significant correlations between NEO-FFI personality traits and setting-specific change scores are shown in Table 4; the full correlation matrix is reported in Supplementary material 1 (Supplementary Table S10). Overall, H3 was not supported, indicating that the preregistered analyses did not provide robust Bonferroni-corrected evidence that personality traits were associated with the magnitude of change across the three measured settings.

**Table 4:** Pearson correlations between NEO-FFI personality traits and setting-specific change scores that reached nominal significance.

Trait	Outcome	Setting	*r*	*p*	pBonf
Openness to experience	Somatic Anxiety (STICSA)	Nature walk-and-talk	−0.211	0.002	0.463
Conscientiousness	Positive Affect (ZIPERS)	Urban walk-and-talk	0.165	0.016	1.000
Agreeableness	Attention (ZIPERS)	Nature walk-and-talk	0.154	0.024	1.000
Neuroticism	Vigor (POMS)	Urban walk-and-talk	0.152	0.027	1.000
Extraversion	Dejection (POMS)	Nature walk-and-talk	−0.151	0.027	1.000
Conscientiousness	Anger (ZIPERS)	Nature walk-and-talk	0.145	0.034	1.000
Conscientiousness	Sadness (ZIPERS)	Urban walk-and-talk	−0.143	0.037	1.000
Extraversion	Diastolic blood pressure	Urban walk-and-talk	−0.142	0.038	1.000
Neuroticism	Attention (ZIPERS)	Indoor	0.142	0.039	1.000
Neuroticism	Diastolic blood pressure	Urban walk-and-talk	0.137	0.046	1.000
Openness to experience	Dejection (POMS)	Nature walk-and-talk	−0.136	0.048	1.000

*N* = 213. NEO-FFI, NEO Five-Factor Inventory; POMS, Profile of Mood States; ZIPERS, Zuckerman Inventory of Personal Reactions; STICSA, State–Trait Inventory for Cognitive and Somatic Anxiety; pBonf, Bonferroni-corrected *p* value. Pearson correlation analyses were conducted between the five NEO-FFI domains and all preregistered setting-specific change scores. Change scores were calculated as post-intervention minus pre-intervention values. Bonferroni-corrected *p* values reflect correction across the 240 preregistered H3 tests (5 personality traits × 48 change scores). No association remained statistically significant after Bonferroni correction.

#### 3.4.2 Environmental identity

Pearson correlation analyses were conducted between Environmental Identity and all setting-specific preregistered change scores. Across the 48 tested associations, 2 correlations reached nominal significance at *p* < 0.05, but none remained significant after Bonferroni correction. The strongest nominal association was observed between Environmental Identity and STICSA Somatic Anxiety change in the nature setting, *r* = −0.170, *p* = 0.0128, pBonf = 0.613. Nominally significant correlations between Environmental Identity and setting-specific change scores are shown in Table 5; the full correlation matrix is reported in Supplementary Material 1 (Supplementary Table S11). Overall, H4 was not supported, indicating that the preregistered analyses did not provide robust Bonferroni-corrected evidence that Environmental Identity was associated with setting-specific change.

**Table 5:** Pearson correlations between environmental identity and setting-specific change scores that reached nominal significance.

Outcome	Setting	*r*	*p*	pBonf
Somatic anxiety (STICSA)	Nature walk-and-talk	−0.170	0.013	0.613
Cognitive anxiety (STICSA)	Nature walk-and-talk	−0.139	0.042	1.000

*N* = 213. STICSA, State–Trait Inventory for Cognitive and Somatic Anxiety; pBonf, Bonferroni-corrected *p* value. Pearson correlation analyses were conducted between Environmental Identity and all preregistered setting-specific change scores. Change scores were calculated as post-intervention minus pre-intervention values. Bonferroni-corrected *p* values reflect correction across the 48 preregistered H4 tests. No association remained statistically significant after Bonferroni correction.

#### 3.4.3 Physical activity

Pearson correlation analyses were conducted between IPAQ total physical activity (MET-min/week) and all setting-specific preregistered change scores. Across the 48 tested associations, 1 correlation reached nominal significance at *p* < 0.05, but it did not remain significant after Bonferroni correction. The strongest observed association was between physical activity and systolic blood pressure change in the indoor setting, *r* = 0.156, *p* = 0.0339, pBonf = 1.000. The nominally significant correlation between total physical activity and setting-specific change is shown in Table 6; the full correlation matrix is reported in Supplementary material 1 (Supplementary Table S12). Overall, H5 was not supported, indicating that the preregistered analyses did not provide robust Bonferroni-corrected evidence that total physical activity was associated with setting-specific change.

**Table 6:** Pearson correlations between total physical activity and setting-specific change scores that reached nominal significance.

Outcome	Setting	*r*	*p*	pBonf
Systolic blood pressure	Indoor	0.156	0.034	1.000

*N* valid = 185, missing = 28. IPAQ, International Physical Activity Questionnaire; MET, metabolic equivalent of task; pBonf, Bonferroni-corrected *p* value. Pearson correlation analyses were conducted between total physical activity (IPAQ total MET-min/week) and all preregistered setting-specific change scores. Change scores were calculated as post-intervention minus pre-intervention values. Bonferroni-corrected *p* values reflect correction across the 48 preregistered H5 tests. Analyses were based on participants with a valid IPAQ score only. No association remained statistically significant after Bonferroni correction.

As an exploratory follow-up to the preregistered H5 analyses, mixed ANOVAs were conducted with setting as a within-subject factor and IPAQ activity level (low, moderate, high) as a between-subject factor. No robust main effects of physical activity level or setting × physical activity level interactions were observed across outcomes; only the interaction for POMS Anger reached nominal significance, *F*(4, 364) = 2.41, *p* = 0.049, *ηp*^2^ = 0.026. Given the exploratory nature of these analyses and the number of tests conducted, this nominal interaction should not be interpreted as robust evidence of moderation by physical activity level (Supplementary material 1: Supplementary Table S13).

### 3.5 Overall summary of preregistered hypotheses

To summarize, the preregistered analyses showed partial support for H1 and support for H2. The strongest Bonferroni-corrected evidence for differences across the three measured sessions was found for POMS Vigor, POMS Dejection, PANAS Negative Affect, systolic blood pressure, diastolic blood pressure, and heart rate. By contrast, no robust Bonferroni-corrected evidence was found for associations of change magnitude with personality traits, environmental identity, or physical activity. Overall, the preregistered results show clearer evidence for differentiated short-term response patterns across the fixed sequence of measured sessions than for individual-difference correlates of change.

## 4 Discussion

The present study examined immediate psychological and physiological responses across a fixed sequence of three psychotherapy/counseling sessions conducted in different socio-physical contexts: an indoor session, an urban walk-and-talk session, and a nature walk-and-talk session. The preregistered analyses showed differentiated short-term response patterns rather than uniform improvement across outcomes or settings. The clearest Bonferroni-corrected psychological differences were observed for POMS Vigor, POMS Dejection, and PANAS Negative Affect, whereas physiological changes were observed in systolic and diastolic blood pressure and heart rate. By contrast, the preregistered individual-difference analyses did not provide robust Bonferroni-corrected evidence that personality traits, Environmental Identity, or physical activity were associated with the magnitude of change.

The results should be interpreted in light of the fixed-order design. Because all participants completed the sessions in the same sequence, setting was fully confounded with session order. The observed differences cannot therefore be uniquely attributed to the environmental setting. They may also reflect therapeutic progression, increasing familiarity with the psychologist or the measurement procedure, adaptation to the study protocol, expectancy effects, carryover between sessions, or changes in the client–psychologist relationship over time. Accordingly, the present results are best understood as preliminary evidence of differentiated response patterns throughout a fixed sequence of indoor, urban walk-and-talk, and nature walk-and-talk sessions, rather than as evidence of isolated causal effects of the socio-physical environment.

Within this interpretive frame, the strongest psychological signal in the present study was observed in the nature-based walk-and-talk session, but only for selected outcomes. Participants showed the largest increase in Vigor and the largest decrease in Dejection in the nature session, whereas Negative Affect decreased similarly in the indoor and nature sessions and less in the urban walk-and-talk session. This pattern does not support the conclusion that the nature setting was globally superior to indoor psychotherapy/counseling. Rather, it suggests that the final session in the fixed sequence, which combined therapeutic interaction, walking, and a predominantly natural environment, was associated with more favorable short-term changes in specific mood-related states. The magnitude and selectivity of these conclusions are important. POMS Vigor showed the clearest difference, whereas several other affective, anxiety-related, and ZIPERS outcomes did not show robust Bonferroni-corrected differences. The psychological findings therefore support a selective, outcome-specific interpretation rather than a broad claim of general psychological benefit.

The observed pattern is broadly compatible with prior qualitative and conceptual work indicating that outdoor talking therapy may be experienced as more spacious, embodied, and affectively facilitative. In their meta-synthesis, Cooley et al. (2021) argued that therapy outdoors may be enriched by mutuality, freedom of expression, mind–body holism, and interconnectedness with nature. Similarly, Meuwese et al. (2021) reported that clients experienced nature during psychotherapy as helping them come closer to their inner being and, in some cases, to the emotional core of their experience. These studies do not directly explain the present quantitative findings, but they do offer a plausible interpretive context for why the nature setting may have been associated with greater Vigor and lower Dejection. Newman and Gabriel (2023) and Greenleaf et al. (2023) similarly emphasized that walk-and-talk may alter the rhythm, informality, and relational positioning of therapy. Such accounts offer a plausible interpretive context for why selected short-term mood states, particularly energy and dejection, may have differed across the measured sessions. However, because the present study did not manipulate environment independently of session order, these qualitative parallels should be understood as interpretive rather than confirmatory. The present data indicate that immediate psychological responses varied across the fixed sequence of sessions, but they do not establish which specific component of the sequence was responsible for the observed differences.

The physiological findings also require careful interpretation. Systolic and diastolic blood pressure increased during the indoor session but decreased slightly in both walk-and-talk sessions, whereas heart rate decreased the most during the indoor session and the least during the nature walk-and-talk session. These findings indicate that short-term bodily responses varied across the three measured sessions, but they should not be interpreted as straightforward evidence of physiological relaxation caused by outdoor or natural environments. The outdoor sessions necessarily involved walking, took place under naturally varying environmental conditions, and occurred within emotionally meaningful psychotherapy/counseling encounters. Although physiological measurements were taken after a standardized resting period, it remains difficult to separate the contributions of environmental exposure, physical movement, emotional engagement, weather, route characteristics, social exposure, and therapeutic interaction. The physiological results are therefore best understood as reflecting psychotherapy/counseling-in-context rather than the independent physiological effect of walking or nature exposure alone.

This pattern is broadly compatible with experimental research showing that walking in forest or greener environments can be associated with greater parasympathetic activity, lower sympathetic activity, lower heart rate, and more favorable affective outcomes than comparison walks in more urban settings (Kobayashi et al., 2018; Lee et al., 2014; Song et al., 2016). At the same time, such comparisons should be made cautiously. The environmental exposure literature typically examines brief walks or viewing tasks rather than counseling or psychotherapy. The present findings, therefore, should not be interpreted as directly confirming or contradicting that literature. Rather, they suggest that the physiological correlates of psychotherapy/counseling-in-context may differ from those observed in non-therapeutic exposure paradigms, likely because psychotherapy and counseling in nature are simultaneously environmental, physical, and emotionally demanding interpersonal processes. Accordingly, the physiological results are best understood as evidence that psychotherapy/counseling-in-context is not reducible to either “walking” or “nature exposure” alone.

Taken together, the psychological and physiological results support a differentiated view of setting-associated responses. The findings do not support a simple model in which nature walk-and-talk uniformly improves psychological and physiological outcomes, nor do they suggest that outdoor sessions can be understood only as walking or nature exposure. Rather, the results are more consistent with the view that psychotherapy/counseling settings may afford different short-term psychological and bodily response patterns depending on the combination of movement, environmental qualities, relational context, emotional engagement, and the therapeutic task. This interpretation is consistent with broader discussion of outdoor therapies, which emphasize promise and clinical relevance while also noting that mechanisms of change remain insufficiently specified (Harper et al., 2021). The present findings therefore contribute to theory development by suggesting that the socio-physical setting of psychotherapy/counseling may be meaningful, but in selective and context-dependent ways.

The individual-difference findings were less robust than the setting-sequence findings. Personality traits, environmental identity, and physical activity did not show Bonferroni-corrected associations with setting-specific change. This result should not be interpreted as definitive evidence that individual differences are irrelevant to walk-and-talk psychotherapy/counseling. Several factors may have contributed to the absence of significant findings. First, the preregistered moderator analyses involved a large number of correlations and used stringent Bonferroni correction, which reduced the likelihood of false-positive findings but increased the risk of Type II error. Second, some measures and administrations showed reduced internal consistency, which may have attenuated associations. Third, the recruitment procedure may have reduced variability in the individual characteristics most relevant to walk-and-talk participation. Clients were approached by their treating psychologists as potentially suitable for the study, agreed to take part in this form of care, and completed all three measured sessions. This may have resulted in a sample with relatively restricted variability in willingness, mobility, openness to outdoor sessions, perceived suitability, and comfort with walk-and-talk work. Such restriction of range may have reduced the likelihood of detecting associations between broad individual-difference variables and setting-specific change.

This consideration is especially relevant for Environmental Identity. Although Environmental Identity is theoretically relevant to nature-related experience, it may have shown limited predictive value in a sample already selected for and willing to participate in walk-and-talk sessions. In such a context, general identification with nature may not be sufficient to differentiate short-term psychological or physiological responses during actual psychotherapy/counseling sessions. More proximal indicators of person-environment fit, such as momentary preference, perceived safety, privacy needs, mobility, comfort with being seen in public, previous experience with outdoor activity, and the therapeutic task of a given session, may be more informative than broad dispositional measures. Contextual fit, session content, and client preference may therefore be more immediate determinants of response than personality traits, environmental identity, or general physical activity level.

The absence of robust individual-difference findings has practical implications. At this stage, the present data do not support identifying an ideal client profile for walk-and-talk psychotherapy/counseling based on personality, environmental identity, or general physical activity level. Decisions about using walk-and-talk formats should therefore remain clinically individualized. Rather than assuming that clients with a particular trait profile will benefit more, psychologists should consider therapeutic goals, client preference, mobility and health status, privacy and confidentiality needs, risk assessment, route suitability, and the fit between the setting and the therapeutic task. This interpretation is consistent with qualitative literature (Cooley et al., 2020; Newman and Gabriel, 2023) emphasizing contracting, contextual suitability, and person-centered decision-making in outdoor therapy rather than a universal indication for all clients.

These conclusions, however, must be interpreted in light of several important limitations. The most important limitation is the fixed order of the three sessions. Although the fixed sequence was chosen to support feasible implementation in routine practice and to introduce outdoor formats gradually, it prevents separation of setting from order-related processes. Future studies should use counterbalanced or randomized setting sequences where ethically and practically feasible or include additional designs capable of distinguishing environmental context from therapeutic progression and habituation.

A second limitation concerns the partially nested structure of the data. Participants were recruited and treated by 24 psychologists, and therapist-level factors may have contributed to the observed response patterns. These factors may include therapeutic orientation, professional experience, interpersonal style, relationship quality, familiarity with walk-and-talk practice, route selection, and differences in implementation. The preregistered repeated-measures ANOVA framework modeled within-person differences across the three measured sessions but did not model therapist-level variance. Because the analytic dataset did not include a reliably usable therapist identifier, therapist-level variance was not estimated statistically. Future research should retain therapist identifiers and use multilevel or other hierarchical approaches to separate participant-level, therapist-level, and setting-related sources of variability.

A third limitation is that the study was embedded in routine psychotherapy/counseling practice rather than designed as a manualized intervention trial. Session content, therapeutic techniques, presenting concerns, emotional intensity, and treatment phase were not systematically standardized or coded. This increases ecological validity, because the study reflects how walk-and-talk may be implemented in real-world care, but it also reduces internal control. Differences between the three measured sessions may partly reflect what was discussed, how emotionally demanding the session was, or how the psychologist adapted the work to the setting. Future studies would benefit from coding session content, therapeutic focus, perceived emotional intensity, and client-rated session depth or helpfulness.

A fourth limitation concerns sample selection and generalizability. Participants were recruited from ongoing care by their treating psychologists, and suitability for participation was determined case by case. The final sample therefore represents clients who were considered suitable for walk-and-talk participation, agreed to take part, and completed the full repeated-measures protocol. It should not be treated as representative of all clients receiving psychotherapy or counseling. Clients with limited mobility, acute clinical instability, health constraints, or strong discomfort with outdoor or public settings may have been less likely to be approached or included. In addition, the sample covered a broad age range from adolescence to older adulthood. Developmental differences in emotional regulation, physiological reactivity, mobility, and comfort with outdoor therapeutic work were not examined. Physiological findings in adolescent participants should also be interpreted with caution because the blood pressure device was validated for adult blood pressure assessment.

A fifth limitation concerns measurement. Several psychological state measures were administered using author-translated Slovak versions, and formal validation of all translated repeated state measures was beyond the scope of the study. In addition, selected administrations showed reduced internal consistency, particularly PANAS Positive Affect in the urban setting, selected POMS administrations, several ZIPERS subscales, and Environmental Identity. These psychometric weaknesses may have attenuated associations and contributed to non-significant findings, especially in the individual-difference analyses. For this reason, findings based on scales with weaker reliability should be interpreted cautiously. Future research should use validated translations where available, further evaluate measurement properties in Slovak samples, and consider repeated-state measures with stronger reliability across multiple administrations.

A sixth limitation concerns environmental characterization. The study distinguished between indoor, urban walk-and-talk, and nature walk-and-talk settings as broad practice contexts, but it did not quantify fine-grained environmental characteristics. Weather and disturbing factors were recorded as contextual information, but variables such as noise level, passers-by density, privacy, perceived safety, route predictability, sensory load, naturalness, and perceived restorativeness were not systematically measured or included as predictors. Consequently, differences between urban and nature walk-and-talk sessions may reflect multiple co-occurring environmental features rather than naturalness alone. Future studies should include more detailed environmental assessment and client-rated perceptions of the setting.

A final statistical consideration concerns the balance between Type I and Type II error. The use of Bonferroni correction was consistent with the preregistered analytic strategy and provided conservative control of false-positive findings across a broad set of outcomes and individual-difference tests. This was appropriate given the number of preregistered comparisons. At the same time, the correction was stringent, particularly for the moderator analyses, and may have reduced sensitivity to small but potentially meaningful associations. The non-significant individual-difference findings should therefore be interpreted as a lack of robust evidence under conservative correction rather than as conclusive evidence that personality, Environmental Identity, and physical activity are unrelated to walk-and-talk responses.

Despite these limitations, the study makes several contributions. It provides quantitative practice-based evidence from a relatively large sample of clients receiving routine psychotherapy/counseling, a field in which empirical evidence remains limited. It also compares the same participants across indoor, urban walk-and-talk, and nature walk-and-talk sessions, thereby reducing interindividual variability in short-term response patterns. Finally, it integrates psychological and physiological outcomes, which allows a broader view of immediate response than self-report measures alone. The study therefore contributes to the developing literature on walk-and-talk psychotherapy/counseling by showing that immediate responses can differ across therapeutic contexts, while also demonstrating that these differences are selective, modest in several cases, and methodologically constrained.

The practical implication of the data is not that indoor psychotherapy or counseling should be replaced by outdoor formats, nor that nature walk-and-talk should be assumed to be superior. Rather, the findings support a more differentiated view. Walk-and-talk, including nature-based walk-and-talk, may represent a viable and potentially useful format within a broader repertoire of psychotherapy/counseling practice, particularly when it fits the client, the therapeutic goals, and the practical context. The setting should be considered as one component of therapeutic planning, alongside confidentiality, safety, accessibility, client preference, route suitability, and the nature of the therapeutic work. In this sense, the present study supports deliberate and context-sensitive use of walk-and-talk rather than generalized claims about its superiority.

The next step for this field is not only to accumulate more studies, but to develop a sharper theory. Future research should build on this work using stronger designs and more detailed measurement. Counterbalanced or randomized setting sequences would be needed to separate environmental setting from session order. Multilevel designs should account for therapist-level clustering and implementation differences. More precise coding of session content, emotional intensity, therapeutic techniques, and environmental characteristics would help clarify mechanisms. Longer follow-up would be needed to determine whether immediate pre-post responses translate into clinically meaningful longer-term outcomes. Finally, future studies should examine more proximal moderators of person-environment fit, including client preference, perceived safety, privacy concerns, mobility, prior experience with nature, and the match between setting and therapeutic task.

In summary, the present study suggests that immediate psychological and physiological responses varied across a fixed sequence of indoor, urban walk-and-talk, and nature walk-and-talk psychotherapy/counseling sessions. The findings point to selective setting-associated response patterns, particularly for Vigor, Dejection, Negative Affect, blood pressure, and heart rate, but they do not establish causal effects of environmental setting alone. The strongest conclusion is therefore not that one setting is universally better, but that the socio-physical context of psychotherapy/counseling deserves explicit attention as a potentially meaningful, context-dependent component of practice and research.

## 5 Conclusion

The present study provides practice-based evidence that immediate psychological and physiological responses varied across a fixed sequence of indoor, urban walk-and-talk, and nature walk-and-talk psychotherapy/counseling sessions. The findings were differentiated rather than uniform. Nature walk-and-talk was associated with more favorable short-term changes in selected psychological outcomes, particularly POMS Vigor and POMS Dejection, whereas physiological responses showed a different pattern, with blood pressure decreasing slightly in both walk-and-talk sessions and heart rate decreasing least in the nature session. Thus, the results do not support a simple conclusion that anyone setting was globally superior across psychological and physiological outcomes.

These conclusions must be interpreted cautiously because setting was confounded with session order. The observed differences cannot be attributed uniquely to environmental context and may also reflect therapeutic progression, increasing familiarity with the psychologist or measurement procedures, adaptation to the study protocol, carryover effects, or other time-related processes. The findings therefore indicate setting-associated response patterns across a fixed sequence of sessions rather than isolated causal effects of indoor, urban, or natural environments.

The study also did not identify robust Bonferroni-corrected individual-difference correlates of setting-specific change. Personality traits, Environmental Identity, and physical activity were not clearly associated with the magnitude of response in the preregistered analyses. These conclusions should not be taken to mean that individual differences are irrelevant, but rather that broad dispositional measures did not provide a reliable basis for identifying who benefits most from walk-and-talk in this practice-based sample.

Overall, the study supports a differentiated and context-sensitive view of walk-and-talk psychotherapy/counseling. Outdoor and nature-based formats should not be treated as replacements for indoor practice or as universally superior options. Rather, they may represent viable additions to psychotherapeutic and counseling practice when they suit the client, the therapeutic goals, and the practical context. The broader implication is that the socio-physical context of psychotherapy/counseling warrants explicit attention in research and practice, and that future counterbalanced, multilevel, and more finely characterized studies are needed to clarify the mechanisms and clinical significance of these immediate response patterns.

## Data Availability

The datasets presented in this study can be found in online repositories. The names of the repository/repositories and accession number(s) can be found at: Open Science Framework at https://osf.io/75qwm/overview?view_only=ef6262ec16124cc3af4a6349fc4887c8.

## Supplementary material

Supplementary Material 1 Scoring, preprocessing, descriptive and psychometric characteristics, and supplementary analyses.

Supplementary Material 2 Paired TOST analyses for setting contrasts.
