# Supplementary material for: From indoor psychotherapy and counseling to walk-and-talk: psychological and physiological responses across a fixed sequence of indoor, urban, and nature-based sessions
Source: Front Psychol. 2026 Aug 13;17:1880259. doi: 10.3389/fpsyg.2026.1880259 (PMC13518116; doi:10.3389/fpsyg.2026.1880259)
Supplement: Supplementary file 1 [file Supplementary_file_1.DOCX]

Supplementary Material 1

Scoring, preprocessing, descriptive statistics, psychometric properties, and supplementary analyses

This supplementary material documents the scoring, preprocessing, naming conventions, descriptive statistics, psychometric properties, and supplementary analyses for the study. Setting codes denote I = indoor setting, II = urban walk-and-talk setting, and III = nature walk-and-talk setting. Unless otherwise stated, all scale scores were computed as mean scores of their constituent items, reverse-coded items were transformed before aggregation, and change scores were calculated as post-intervention minus pre-intervention values. Positive change scores indicate an increase from pre- to post-intervention, whereas negative change scores indicate a decrease. Non-rounded values were retained in the analytic dataset to preserve analytical precision, whereas values in tables are rounded for presentation.

1 **General scoring and preprocessing procedures**
  1.1 Setting codes and change-score definition

Setting codes were used to identify the three intervention contexts in the analytic dataset. Code I refers to the indoor setting, code II to the urban walk-and-talk setting, and code III to the nature walk-and-talk setting. Across all repeated measures, pre indicates the pre-intervention assessment, post indicates the post-intervention assessment, and change indicates the change score calculated as post-intervention minus pre-intervention.

| **Code or suffix** | **Meaning in the dataset** |
| --- | --- |
| I, II, III | Intervention setting (I = indoor; II = urban walk-and-talk; III = nature walk-and-talk) |
| pre | Pre-intervention score |
| post | Post-intervention score |
| change | Change score computed as post - pre |

1.2 **Dataset naming conventions**

The analytic dataset used standardized construct names with scale prefixes where needed to avoid ambiguity. PANAS variables were labeled as panas_positive_affect and panas_negative_affect; POMS-16 variables as poms_vigor, poms_anger, poms_fatigue, and poms_dejection; ZIPERS variables as zipers_positive_affect, zipers_attention, zipers_fear, zipers_sadness, and zipers_anger; STICSA variables as sticsa_cognitive_anxiety and sticsa_somatic_anxiety; and physiological variables as systolic_bp, diastolic_bp, and heart_rate. Setting-specific variables were indexed by setting code or setting name, and pre, post, and change/difference suffixes indicated the measurement phase or change score.

1.3 **General scoring principles**

Unless otherwise stated, all scale scores reported in this supplementary material were computed as mean scores of their constituent items. Reverse-coded items were transformed prior to score aggregation. For repeated pre- and post-intervention assessments, change scores were calculated uniformly as post-intervention minus pre-intervention values, so that positive values indicate an increase and negative values indicate a decrease from pre- to post-intervention. Non-rounded values were retained in the analytic dataset where necessary to preserve analytical precision, although values are rounded for presentation in the tables.

1.4 **Psychometric indices**

Internal consistency was evaluated using Cronbach’s alpha for repeated state measures and using Cronbach’s alpha and McDonald’s omega for selected baseline individual-difference measures. Reliability indices are reported separately for each administration where applicable, because repeated state measures were administered across multiple settings and time points. For single-item indicators, internal consistency was not applicable. Very low or negative reliability coefficients were interpreted as evidence that the corresponding items did not function consistently in that specific administration and are discussed as psychometric limitations.

1.5 **Missing data and precision of stored values**

Missing data were handled according to the analytic strategy described in the main manuscript. Primary repeated-measures analyses were based on participants with complete repeated psychological and physiological change scores across all three settings. Analyses involving physical activity were based only on participants with valid IPAQ-derived total MET-min/week values. Non-rounded values were retained in the analytic dataset for all computed scale and change scores, whereas values are rounded in the supplementary tables for presentation.

2 **Measure-specific scoring**
  2.1 **PANAS**

The Positive and Negative Affect Schedule (PANAS; Watson et al., 1988) was processed separately for settings I, II, and III. For each setting, Positive Affect and Negative Affect were each calculated as the mean of 10 items scored on a 1-5 response scale. For both dimensions, pre, post, and change scores were stored, with change defined as post - pre.

2.2 **POMS-16**

The Profile of Mood States, 16-item version (POMS-16; Petrowski et al., 2021; based on McNair et al., 1971), was scored for four subscales: Vigor, Anger, Fatigue, and Dejection. Each subscale was calculated as the mean of four items scored on a 0–4 response scale. Pre-intervention, post-intervention, and change scores were derived for each setting, with change defined as post-intervention minus pre-intervention.

2.3 **ZIPERS**

The Zuckerman Inventory of Personal Reactions (ZIPERS; Zuckerman, 1977) variables were scored for five subscales: Positive Affect, Attention, Fear, Sadness, and Anger. Positive Affect was calculated as the mean of four items, Attention as the mean of two items, Fear as the mean of three items, Sadness as a single-item score, and Anger as the mean of two items. Items used a 1-5 response scale. One item in the Attention subscale was reverse-scored before aggregation using the standard transformation (reversed score = scale maximum + scale minimum - original score). Pre, post, and change scores were stored for each setting, with change defined as post - pre.

2.4 **STICSA**

The State-Trait Inventory for Cognitive and Somatic Anxiety (STICSA; Ree et al., 2008), was scored for two “state” dimensions: Cognitive Anxiety (mean of 11 items) and Somatic Anxiety (mean of 10 items). Items were scored on a 1-4 response scale. Pre, post, and change scores were computed for each setting, with change defined as post - pre.

2.5 **Physiological measures**

Physiological outcomes included systolic blood pressure, diastolic blood pressure, and heart rate measured before and after the intervention in each setting. Blood pressure and heart rate were measured using the Huawei Watch D. For blood pressure measurement, the Huawei Watch D has been validated for adult blood pressure assessment according to the ANSI/AAMI/ISO 81060-2:2018 standard and is listed as a validated home blood pressure monitor in the STRIDE BP database. Because the device has been validated for adult blood pressure assessment, readings from adolescent participants should be interpreted with additional caution. Physiological measurements were obtained according to a standardized protocol with participants in a seated position after 5 minutes of rest. For outdoor sessions, measurements were taken outdoors using the same procedure before and after each session. Heart rate was measured twice at each assessment point, and the session value was calculated as the mean of the two readings. For blood pressure, two readings were obtained routinely at each time point. A third reading was taken only when the discrepancy between the first and second measurements exceeded 10 mmHg for systolic blood pressure or 6 mmHg for diastolic blood pressure. Session values for systolic and diastolic blood pressure were then calculated as the mean of the available readings. For each physiological variable, change scores were computed as post-intervention minus pre-intervention values.

2.6 **NEO-FFI**

Personality dimensions were derived from the NEO Five-Factor Inventory (NEO-FFI), yielding the domains Neuroticism, Extraversion, Openness to Experience, Agreeableness, and Conscientiousness. Each domain comprised 12 items scored on a 0-4 response scale. Reverse-keyed items were reverse-scored before aggregation, and final domain scores were computed as the mean of the 12 scored items.

2.7 **Environmental Identity**

Environmental identity was assessed using the revised Environmental Identity Scale (Clayton et al., 2021). The version used in the present study comprised 14 items scored on a 1–7 response scale. The total score (environmental_identity_total) was calculated as the mean of all 14 items, with higher scores indicating stronger environmental identity. Scores were retained in non-rounded form to preserve analytical precision.

2.8 **IPAQ**

Physical activity was assessed using the International Physical Activity Questionnaire, Short Form (IPAQ-SF). Derived variables represented weekly frequency (days/week) and daily duration (minutes/day) of vigorous activity, moderate activity, and walking.

Preprocessing

- Days/week values were restricted to the range 0-7.
- Minutes/day values below 10 were treated as missing, and values above 180 were truncated to 180.
- Non-informative responses (e.g., “I don’t know”) were treated as missing values.
- If either the number of days or the number of minutes was missing for a given activity type, the corresponding activity component was coded as missing.

*MET-minute calculation and classification*

Total physical activity was expressed in MET-minutes per week (MET-min/week) using standard IPAQ coefficients: vigorous activity = 8.0 METs, moderate activity = 4.0 METs, and walking = 3.3 METs. Component scores were calculated as days × minutes × coefficient and summed to create ipaq_total_met. If all three components were missing, the total score was coded as missing.

Participants were additionally classified in ipaq_level as low (<600 MET-min/week), moderate (600-2999 MET-min/week), or high (>=3000 MET-min/week). A missing-data approach was used for the primary scoring. Sensitivity analyses using zero-imputation yielded comparable distributional patterns but slightly lower mean activity levels.

3 **Descriptive and psychometric results**

Omnibus ANOVA results in this section are reported to align the descriptive and psychometric information with the preregistered analyses presented in the main manuscript. Where relevant, Greenhouse–Geisser-corrected degrees of freedom and p values are reported. Bonferroni-corrected omnibus p values for the primary preregistered tests are reported in the main manuscript.

3.1 **Positive and Negative Affect Schedule (PANAS)**

The Positive and Negative Affect Schedule (PANAS) was scored separately for each setting and time point. Positive Affect and Negative Affect scores were calculated as mean scores of 10 items each, and change scores were defined as post-intervention minus pre-intervention values. Complete PANAS-derived scores were available for all participants. Internal consistency for Positive Affect was acceptable to good in the indoor and nature-based administrations (Cronbach’s αs = .801-.881), but substantially lower in the urban administration (pre: α = .228; post: α = .451). Negative Affect showed acceptable to good internal consistency across administrations (αs = .675-.874). Mean Positive Affect increased in all three settings (indoor: *M* = 0.23, *SD* = 0.66; urban walk-and-talk: *M* = 0.35, *SD* = 0.49; nature walk-and-talk: *M* = 0.26, *SD* = 0.68), whereas Negative Affect decreased in all three settings (indoor: *M* = -0.32, *SD* = 0.54; urban walk-and-talk: *M* = -0.14, *SD* = 0.73; nature walk-and-talk: *M* = -0.32, *SD* = 0.56). Repeated-measures analyses indicated an effect of setting for Positive Affect, *F*(1.55, 329.58) = 4.12, *p* = .026, η² = .007, and Negative Affect, *F*(1.17, 246.99) = 8.55, *p* = .002, η² = .020. Because the urban Positive Affect administration showed poor internal consistency, findings involving this score should be interpreted with caution.

Table S1: PANAS descriptive statistics and internal consistency across settings

| **Scale** | **Setting** | **Pre M (SD)** | **Post M (SD)** | **Change M (SD)** | **Change Min** | **Change**  **Max** | **Cronbach’s α pre** | **Cronbach’s α post** |
| --- | --- | --- | --- | --- | --- | --- | --- | --- |
| Positive Affect | Indoor | 2.93 (0.69) | 3.16 (0.85) | 0.23 (0.66) | -1.40 | 2.10 | .801 | .876 |
|  | Urban walk-and-talk | 3.02 (0.33) | 3.37 (0.39) | 0.35 (0.49) | -1.00 | 2.00 | .228 | .451 |
|  | Nature walk-and-talk | 2.89 (0.72) | 3.15 (0.85) | 0.26 (0.68) | -0.90 | 2.40 | .827 | .881 |
| Negative Affect | Indoor | 1.94 (0.51) | 1.62 (0.60) | -0.32 (0.54) | -1.70 | 1.70 | .675 | .861 |
|  | Urban walk-and-talk | 1.80 (0.46) | 1.66 (0.46) | -0.14 (0.73) | -1.00 | 1.00 | .686 | .724 |
|  | Nature walk-and-talk | 1.93 (0.50) | 1.61 (0.61) | -0.32 (0.56) | -1.50 | 1.70 | .683 | .874 |

**Note.** N = 213. Positive Affect and Negative Affect scores were computed as mean scores of 10 items each. Change scores were calculated as post-intervention minus pre-intervention values.

A notable psychometric issue was observed for the Positive Affect scale in the urban walk-and-talk administration, which showed poor internal consistency at both pre- and post-intervention assessment (α = .228 and .451, respectively), accompanied by very low mean inter-item correlations and several near-zero or negative corrected item-total correlations. In addition, the urban Positive Affect scores showed restricted variability and elevated kurtosis. Findings involving this score should therefore be interpreted with caution.

**3.2 Profile of Mood States (POMS-16)**

The Profile of Mood States (POMS-16) was scored separately for each setting and time point. Four subscales were analyzed: Vigor, Anger, Fatigue, and Dejection. Each subscale score was calculated as the mean of four items, and change scores were defined as post-intervention minus pre-intervention values. Complete POMS-derived scores were available for all participants (N = 213). Vigor increased across all three settings, with the largest increase observed in the nature walk-and-talk condition (indoor: *M* = 0.16, *SD* = 0.72; urban walk-and-talk: *M* = 0.26, *SD* = 0.61; nature walk-and-talk: *M* = 1.05, *SD* = 1.37). Dejection decreased in all three settings, with the largest decrease again observed in the nature walk-and-talk condition (indoor: *M* = -0.29, *SD* = 0.50; urban walk-and-talk: *M* = -0.29, *SD* = 0.54; nature walk-and-talk: *M* = -0.52, *SD* = 0.74). Repeated-measures analyses indicated a significant effect of setting for Vigor, *F*(1.53, 324.68) = 53.50, *p* < .001, η² = .146, and Dejection, *F*(1.89, 399.63) = 10.11, *p* < .001, η² = .031, whereas Anger, *F*(1.89, 400.33) = 0.92, *p* = .396, η² = .003, and Fatigue, *F*(1.98, 419.11) = 0.03, *p* = .966, η² < .001, did not differ across settings. Internal consistency was acceptable to excellent for most indoor and urban administrations, but several nature-based administrations showed poor internal consistency, particularly for Anger, Fatigue, and Dejection; findings involving these scores should therefore be interpreted with caution.

Table S2: POMS descriptive statistics and internal consistency across settings

| **Scale** | **Setting** | **Pre M (SD)** | **Post M (SD)** | **Change M (SD)** | **Change Min** | **Change**  **Max** | **Cronbach’s α pre** | **Cronbach’s α post** |
| --- | --- | --- | --- | --- | --- | --- | --- | --- |
| Vigor | Indoor | 1.83 (0.76) | 1.99 (0.95) | 0.16 (0.72) | -1.00 | 2.75 | .830 | .920 |
|  | Urban walk-and-talk | 1.80 (0.75) | 2.06 (0.87) | 0.26 (0.61) | -1.25 | 1.25 | .791 | .858 |
|  | Nature walk-and-talk | 1.37 (0.74) | 2.42 (0.90) | 1.05 (1.37) | -1.50 | 3.25 | .660 | .768 |
| Anger | Indoor | 0.40 (0.49) | 0.21 (0.48) | -0.19 (0.38) | -1.50 | 0.50 | .670 | .861 |
|  | Urban walk-and-talk | 0.47 (0.74) | 0.23 (0.41) | -0.25 (0.53) | -1.50 | 1.25 | .898 | .822 |
|  | Nature walk-and-talk | 0.61 (0.33) | 0.36 (0.34) | -0.25 (0.46) | -2.00 | 1.75 | -.267 | .270 |
| Fatigue | Indoor | 2.05 (1.09) | 1.48 (1.07) | -0.57 (0.70) | -2.50 | 0.75 | .897 | .917 |
|  | Urban walk-and-talk | 1.82 (1.23) | 1.24 (1.14) | -0.58 (0.76) | -2.50 | 1.00 | .934 | .928 |
|  | Nature walk-and-talk | 2.20 (0.58) | 1.61 (0.61) | -0.59 (0.71) | -2.00 | 1.75 | .260 | .378 |
| Dejection | Indoor | 1.06 (0.98) | 0.77 (0.91) | -0.29 (0.50) | -2.50 | 0.75 | .868 | .900 |
|  | Urban walk-and-talk | 1.00 (0.94) | 0.71 (0.82) | -0.29 (0.54) | -3.00 | 1.00 | .895 | .901 |
|  | Nature walk-and-talk | 1.29 (0.59) | 0.77 (0.41) | -0.52 (0.74) | -2.25 | 1.00 | .321 | -.015 |

**Note.** N = 213. Each POMS subscale was computed as the mean of four items. Cronbach’s α values are reported separately for pre- and post-intervention administrations.

Several POMS administrations in the nature walk-and-talk condition showed poor internal consistency, particularly for Anger (pre: α = -.267; post: α = .270), Fatigue (pre: α = .260; post: α = .378), and Dejection (post: α = -.015). Very low or negative alpha coefficients indicate that the items within these four-item subscales did not function consistently in this administration and should not be interpreted as evidence of reliable scale measurement. Accordingly, findings involving these specific scores should be interpreted with caution, and greater weight should be given to results from subscales and administrations showing acceptable internal consistency. This limitation is particularly relevant for the interpretation of the POMS Dejection finding in the nature walk-and-talk condition, because the nature post-intervention Dejection administration showed very low internal consistency. The statistically robust setting-related finding for POMS Dejection should therefore be interpreted cautiously as a short-term scale-score pattern rather than as strong evidence of reliable measurement at that specific administration.

3.3 **Zuckerman Inventory of Personal Reactions (ZIPERS)**

The Zuckerman Inventory of Personal Reactions (ZIPERS) was scored separately for each setting and time point. Five dimensions were analyzed: Positive Affect, Attention, Fear, Sadness, and Anger. Positive Affect was calculated as the mean of four items, Attention as the mean of two items including one reverse-coded item, Fear as the mean of three items, Sadness as a single-item indicator, and Anger as the mean of two items. Change scores were defined as post-intervention minus pre-intervention values. Complete ZIPERS-derived scores were available for all participants (*N* = 213). Across settings, ZIPERS Positive Affect and Attention increased, whereas Fear, Sadness, and Anger decreased after the intervention. However, repeated-measures analyses indicated no robust setting-related differences for ZIPERS Positive Affect, F(1.79, 379.29) = 1.28, p = .276, η² = .004; ZIPERS Attention, F(1.92, 406.74) = 1.88, p = .156, η² = .006; ZIPERS Fear, F(1.54, 327.24) = 0.41, p = .608, η² = .001; ZIPERS Sadness, F(1.60, 338.33) = 0.86, p = .402, η² = .003; or ZIPERS Anger, F(1.75, 370.72) = 0.13, p = .855, η² < .001. Greenhouse–Geisser-corrected degrees of freedom and p values are reported where applicable, consistent with the main manuscript.

Table S3: ZIPERS descriptive statistics and internal consistency across settings

| **Scale** | **Setting** | **Pre M (SD)** | **Post M (SD)** | **Change M (SD)** | **Change Min** | **Change**  **Max** | **Cronbach’s α pre** | **Cronbach’s α post** |
| --- | --- | --- | --- | --- | --- | --- | --- | --- |
| Positive Affect | Indoor | 2.93 (0.81) | 3.34 (0.89) | 0.41 (0.72) | -1.25 | 2.25 | .807 | .868 |
|  | Urban walk-and-talk | 2.88 (0.42) | 3.33 (0.46) | 0.45 (0.52) | -1.25 | 2.25 | -.246 | -.070 |
|  | Nature walk-and-talk | 2.88 (0.40) | 3.38 (0.43) | 0.50 (0.49) | -1.00 | 2.50 | -.413 | -.082 |
| Attention | Indoor | 3.75 (0.80) | 3.90 (0.72) | 0.14 (0.81) | -2.00 | 2.00 | .359 | .016 |
|  | Urban walk-and-talk | 3.62 (0.48) | 3.94 (0.90) | 0.32 (0.96) | -2.50 | 2.50 | -3.582 | .240 |
|  | Nature walk-and-talk | 3.89 (0.85) | 4.08 (0.72) | 0.19 (1.12) | -3.00 | 3.50 | .366 | -.078 |
| Fear | Indoor | 1.78 (0.85) | 1.56 (0.54) | -0.22 (0.86) | -2.33 | 2.00 | .790 | .408 |
|  | Urban walk-and-talk | 1.85 (0.89) | 1.63 (0.69) | -0.23 (1.19) | -3.00 | 3.00 | .710 | .658 |
|  | Nature walk-and-talk | 1.67 (0.49) | 1.38 (0.35) | -0.29 (0.61) | -1.67 | 1.00 | .153 | .054 |
| Sadness | Indoor | 2.34 (1.18) | 1.72 (0.90) | -0.62 (1.33) | -4.00 | 3.00 | - | - |
|  | Urban walk-and-talk | 2.22 (0.62) | 1.67 (0.62) | -0.54 (0.66) | -2.00 | 1.00 | - | - |
|  | Nature walk-and-talk | 2.38 (0.64) | 1.88 (0.57) | -0.50 (0.72) | -3.00 | 1.00 | - | - |
| Anger | Indoor | 1.41 (0.83) | 1.21 (0.50) | -0.20 (0.83) | -4.00 | 1.50 | .827 | .663 |
|  | Urban walk-and-talk | 1.50 (0.58) | 1.27 (0.27) | -0.23 (0.63) | -2.00 | 0.50 | .270 | -.672 |
|  | Nature walk-and-talk | 1.49 (0.40) | 1.29 (0.30) | -0.20 (0.45) | -1.00 | 0.50 | -.343 | -.596 |

**Note.** N = 213. ZIPERS subscales were computed as follows: Positive Affect = mean of four items, Attention = mean of two items, Fear = mean of three items, Sadness = single-item indicator, and Anger = mean of two items. Cronbach’s α values are not applicable to Sadness.

Several ZIPERS administrations showed poor internal consistency, particularly in the urban and nature walk-and-talk conditions. The most problematic results were observed for Positive Affect (urban pre: α = -.246, urban post: α = -.070, nature pre: α = -.413, nature post: α = -.082), Attention (indoor pre: α = .359, indoor post: α = .016, urban pre: α = -3.582, urban post: α = .240, nature pre: α = .366, nature post: α = -.078), and Anger (urban pre: α = .270, urban post: α = -.672, nature pre: α = -.343, nature post: α = -.596). Fear also showed weak reliability in the nature condition (pre: α = .153, post: α = .054). Negative or very low alpha coefficients indicate that the items within these subscales did not function consistently in these administrations and should be interpreted as a psychometric limitation rather than as evidence of reliable scale measurement. In addition, Sadness was assessed as a single-item indicator, and internal consistency is therefore not applicable. Accordingly, findings involving these ZIPERS scores should be interpreted with caution. Given these psychometric limitations, ZIPERS-derived findings should be treated as descriptive and interpreted with less weight than findings from measures and administrations with acceptable internal consistency.

3.4 **The State-Trait Inventory for Cognitive and Somatic Anxiety (STICSA)**

The State-Trait Inventory for Cognitive and Somatic Anxiety (STICSA) was scored separately for each setting and time point. Two domains were analyzed: Cognitive Anxiety, calculated as the mean of 11 items, and Somatic Anxiety, calculated as the mean of 10 items. Change scores were defined as post-intervention minus pre-intervention values. Complete STICSA-derived scores were available for all participants (*N* = 213). Cognitive Anxiety decreased across all three settings (indoor: *M* = -0.35, *SD* = 0.33; urban walk-and-talk: *M* = -0.37, *SD* = 0.44; nature walk-and-talk: *M* = -0.36, *SD* = 0.44), as did Somatic Anxiety (indoor: *M* = -0.16, *SD* = 0.30; urban walk-and-talk: *M* = -0.11, *SD* = 0.34; nature walk-and-talk: *M* = -0.11, *SD* = 0.23). Repeated-measures analyses indicated no robust setting-related differences for Cognitive Anxiety, F(1.96, 414.69) = 0.29, p = .741, η² = .001, or Somatic Anxiety, F(1.85, 392.42) = 1.91, p = .153, η² = .006. Internal consistency was generally acceptable to good across administrations, although the indoor pre-intervention administrations showed comparatively lower reliability, particularly for Somatic Anxiety (*α* = .583) and, to a lesser extent, Cognitive Anxiety (*α* = .659).

Table S4: STICSA descriptive statistics and internal consistency across settings

| **Scale** | **Setting** | **Pre M (SD)** | **Post M (SD)** | **Change M (SD)** | **Change Min** | **Change**  **Max** | **Cronbach’s α pre** | **Cronbach’s α post** |
| --- | --- | --- | --- | --- | --- | --- | --- | --- |
| Cognitive Anxiety | Indoor | 2.34 (0.48) | 1.99 (0.55) | -0.35 (0.33) | -1.55 | 0.46 | .659 | .796 |
|  | Urban walk-and-talk | 2.34 (0.67) | 1.97 (0.71) | -0.37 (0.44) | -2.09 | 0.09 | .844 | .895 |
|  | Nature walk-and-talk | 2.27 (0.60) | 1.91 (0.65) | -0.36 (0.44) | -1.82 | 0.46 | .816 | .881 |
| Somatic Anxiety | Indoor | 1.54 (0.36) | 1.39 (0.39) | -0.16 (0.30) | -1.10 | 0.70 | .583 | .766 |
|  | Urban walk-and-talk | 1.44 (0.51) | 1.33 (0.49) | -0.11 (0.34) | -1.40 | 0.50 | .863 | .890 |
|  | Nature walk-and-talk | 1.40 (0.46) | 1.29 (0.42) | -0.11 (0.23) | -1.40 | 0.40 | .833 | .859 |

**Note.** N = 213. Cognitive Anxiety was computed as the mean of 11 items, and Somatic Anxiety as the mean of 10 items. Change scores were calculated as post-intervention minus pre-intervention values.

Overall, the STICSA administrations showed acceptable to good internal consistency across settings. The main psychometric limitation was observed in the indoor pre-intervention administration, where reliability was comparatively lower for Cognitive Anxiety (α = .659) and especially for Somatic Anxiety (α = .583). Although these coefficients do not indicate severe scale failure, they suggest reduced internal consistency relative to the other administrations. Findings involving these specific scores should therefore be interpreted with somewhat greater caution than results from the urban and nature-based administrations, which showed stronger reliability.

3.5 **Physiological measures**

Physiological measures included systolic blood pressure, diastolic blood pressure, and heart rate assessed before and after the intervention in each setting. Heart rate was measured twice at each time point, and the session value was calculated as the mean of the two readings. For blood pressure, two readings were obtained routinely at each time point. A third reading was taken only when the discrepancy between the first and second measurements exceeded the predefined threshold, that is, > 10 mmHg for systolic blood pressure or > 6 mmHg for diastolic blood pressure. Session values for systolic and diastolic blood pressure were then calculated as the mean of the available readings. Change scores were defined uniformly as post-intervention minus pre-intervention values.

Complete derived scores were available for all participants (N = 213). Descriptive statistics showed that indoor sessions were associated with increases in systolic blood pressure (M = 3.77, SD = 13.53) and diastolic blood pressure (M = 1.11, SD = 5.17), whereas urban and nature walk-and-talk sessions were associated with small decreases in both blood pressure indices. Heart rate decreased across all three settings, with the largest reduction observed in the indoor condition (M = -3.49, SD = 10.30). Repeated-measures analyses of variance indicated significant Bonferroni-corrected setting-related differences for systolic blood pressure, F(1.35, 285.37) = 15.77, p < .001, η² = .047, pBonf < .001; diastolic blood pressure, F(1.97, 417.53) = 10.74, p < .001, η² = .031, pBonf = .001; and heart rate, F(1.91, 404.64) = 6.98, p = .001, η² = .019, pBonf = .020. Pairwise comparisons showed that the indoor setting differed significantly from both walk-and-talk settings for systolic and diastolic blood pressure, whereas for heart rate the nature walk-and-talk setting differed significantly from both the indoor and urban settings.

Table S5: Descriptive statistics for physiological measures across settings

| **Measure** | **Setting** | **Pre M (SD)** | **Post M (SD)** | **Change M (SD)** | **Change Min** | **Change**  **Max** | **Change Skewness** | **Change Kurtosis** |
| --- | --- | --- | --- | --- | --- | --- | --- | --- |
| Systolic blood pressure | Indoor | 114.77 (9.19) | 118.55 (12.03) | 3.77 (13.53) | -33.50 | 43.00 | -0.10 | 0.23 |
|  | Urban walk-and-talk | 117.81 (6.34) | 116.85 (6.30) | -0.95 (6.08) | -22.33 | 14.00 | -0.47 | -0.15 |
|  | Nature walk-and-talk | 119.35 (6.85) | 118.87 (8.81) | -0.48 (7.64) | -30.50 | 28.67 | 0.09 | 2.08 |
| Diastolic blood pressure | Indoor | 77.28 (5.79) | 78.40 (4.79) | 1.11 (5.17) | -20.33 | 26.50 | -0.09 | 3.27 |
|  | Urban walk-and-talk | 78.90 (4.30) | 77.78 (4.81) | -1.12 (5.36) | -13.50 | 10.83 | 0.11 | -0.36 |
|  | Nature walk-and-talk | 79.66 (4.27) | 78.79 (5.71) | -0.87 (6.13) | -10.50 | 15.33 | 0.16 | -0.86 |
| Heart rate | Indoor | 78.15 (9.39) | 74.67 (6.28) | -3.49 (10.30) | -25.00 | 42.00 | 1.66 | 6.11 |
|  | Urban walk-and-talk | 82.88 (5.78) | 80.18 (5.77) | -2.70 (6.25) | -20.50 | 11.00 | -0.18 | -0.05 |
|  | Nature walk-and-talk | 80.29 (5.75) | 79.59 (6.99) | -0.70 (8.02) | -16.00 | 23.00 | 0.29 | 0.18 |

**Note.** N = 213. Blood pressure values (mmHg) were computed as the mean of two or three repeated readings per phase; heart rate values (bpm) were computed as the mean of two repeated readings per phase. Change scores were calculated as post-intervention minus pre-intervention values.

All derived physiological variables were available for the full sample (N = 213). No values were identified as physiologically impossible or clearly indicative of data entry error. However, several variables showed noticeable departures from normality, particularly urban pre- and post-session systolic blood pressure, nature post-session systolic blood pressure, indoor post-session heart rate, and indoor heart rate change scores, which displayed elevated skewness and kurtosis. In addition, a relatively high number of IQR-based outliers was observed for some physiological variables, especially heart rate and selected systolic and diastolic blood pressure indices. These values were retained because they fell within plausible ranges and likely reflect genuine inter-individual variability rather than measurement error. The physiological findings should thus be interpreted with appropriate caution, particularly for variables showing stronger distributional asymmetry or a higher concentration of outlying observations.

3.6 **NEO Five-Factor Inventory (NEO-FFI)**

Supplementary Table S6 presents detailed psychometric properties of the NEO Five-Factor Inventory (NEO-FFI) in the present sample. The file includes scale-level descriptive statistics, internal consistency indices (Cronbach’s alpha and McDonald’s omega), item-level statistics, inter-scale correlations, and the scoring key with reverse-coded items. NEO-FFI domain scores were calculated as mean scores across 12 items per domain after reverse coding, with possible scores ranging from 0 to 4. This supplementary material is provided to document the reliability and scoring characteristics of the instrument in the current study sample.

Table S6: Descriptive Statistics for NEO-FFI.

| **Domain** | **M** | **SD** | **Min** | **Max** | **Skewness** | **Kurtosis** | **Cronbach’s α**  **(95% CI)** | **McDonald’s ωt (95% CI)** |
| --- | --- | --- | --- | --- | --- | --- | --- | --- |
| Neuroticism | 2.19 | 0.82 | 1.00 | 3.75 | 0.02 | -1.45 | .906 [.888, .922] | .911 [.893, .926] |
| Extraversion | 2.10 | 0.51 | 0.25 | 3.17 | 0.10 | 0.17 | .703 [.629, .757] | .776 [.727, .810] |
| Openness to Experience | 2.27 | 0.51 | 0.75 | 3.42 | -1.67 | 3.77 | .615 [.428, .708] | .560 [.406, .699] |
| Agreeableness | 2.25 | 0.60 | 0.58 | 3.50 | -0.29 | -0.92 | .858 [.835, .877] | .806 [.761, .856] |
| Conscientiousness | 2.85 | 0.40 | 1.50 | 3.83 | 0.43 | 0.52 | .714 [.640, .763] | .653 [.493, .729] |

**Note.** N = 213. Domain scores were calculated as mean scores across 12 items after reverse coding, with possible scores ranging from 0 to 4.

Inspection of distributional indices indicated that Openness to Experience showed the largest deviation from normality (skewness = -1.67, kurtosis = 3.77), whereas the remaining NEO-FFI dimensions showed only minor to moderate deviations. Openness to Experience also showed lower internal consistency than the other NEO-FFI domains. Accordingly, nominal associations involving this domain should be interpreted with caution, particularly given that no personality-related association remained significant after Bonferroni correction.

3.7 **Environmental Identity**

Environmental identity was assessed using a 14-item scale, and the total score was calculated as the mean of all items, with higher scores indicating stronger environmental identity. In the present sample the Environmental Identity total score showed a mean of 4.90 (SD = 0.52), with observed scores ranging from 3.79 to 6.71. Distributional indices indicated moderate positive skewness (0.69) and elevated kurtosis (1.40). Internal consistency was modest, with Cronbach’s α = .578, 95% CI [.439, .670], and McDonald’s ω = .598, 95% CI [.331, .687].

Table S7: Psychometric properties of the Environmental Identity scale in the present sample

| **Scale** | **M** | **SD** | **Min** | **Max** | **Skewness** | **Kurtosis** | **Cronbach’s α**  **(95% CI)** | **McDonald’s ωt (95% CI)** |
| --- | --- | --- | --- | --- | --- | --- | --- | --- |
| Environmental Identity | 4.90 | 0.52 | 3.79 | 6.71 | 0.69 | 1.40 | .578 [.439, .670] | .598 [.331, .687] |

**Note.** N = 213. The Environmental Identity total score was computed as the mean of 14 items. Higher scores indicate stronger environmental identity.

Environmental Identity showed modest internal consistency in the present sample (α = .578, ωt = .598). Several items displayed weak item-total correlations, and one item showed a restricted response range, suggesting that findings involving this measure should be interpreted with caution. This psychometric limitation should be considered when interpreting the preregistered H4 analyses involving Environmental Identity, particularly because no association remained significant after correction for multiple testing.

3.8 **IPAQ total physical activity — Table S8**

**International Physical Activity Questionnaire (IPAQ)**

Physical activity was assessed using International Physical Activity Questionnaire (IPAQ)-derived variables representing weekly frequency (days/week) and daily duration (minutes/day) of vigorous activity, moderate activity, and walking. Scoring followed a missing-data approach: days were restricted to the range 0-7, minutes below 10 per day were treated as missing, and values above 180 minutes/day were truncated to 180. Non-informative responses were treated as missing. Total physical activity was expressed as MET-min/week using standard IPAQ coefficients (8.0 for vigorous activity, 4.0 for moderate activity, and 3.3 for walking). In the present sample, IPAQ total physical activity showed a mean of 2369.9 MET-min/week (SD = 1934.4), a median of 1973.0, and a range from 0 to 10080, based on 185 valid cases, with 28 cases missing. Distributional indices indicated substantial positive skewness (1.284) and elevated kurtosis (2.030), suggesting a non-normal distribution. Activity level classification showed that 33 participants (15.5%) were categorized as low, 96 (45.1%) as moderate, and 56 (26.3%) as high, whereas 28 participants (13.1%) had missing IPAQ classification.

Table S8: Descriptive statistics of IPAQ total physical activity (missing-data approach)

| **Variable** | **M** | **SD** | **Min** | **Max** | **Skewness** | **Kurtosis** |
| --- | --- | --- | --- | --- | --- | --- |
| IPAQ total physical activity (MET-min/week) | 2369.9 | 1934.4 | 0 | 10080 | 1.284 | 2.03 |

**Note.** N valid = 185, missing = 28. Total physical activity was calculated in MET-min/week using standard IPAQ coefficients (8.0 for vigorous activity, 4.0 for moderate activity, and 3.3 for walking) and a missing-data approach. Days were restricted to 0–7, values below 10 minutes/day were treated as missing, and values above 180 minutes/day were truncated to 180. Statistics are based on valid IPAQ cases only.

3.9 **IPAQ activity levels — Table S9**

Table S9: Distribution of IPAQ activity levels (missing-data approach)

| IPAQ level | **N** | Percent of total sample | Percent of valid IPAQ cases |
| --- | --- | --- | --- |
| Low | 33 | 15.5 | 17.8 |
| Moderate | 96 | 45.1 | 51.9 |
| High | 56 | 26.3 | 30.3 |
| Missing | 28 | 13.1 | - |

**Note.** Activity levels were classified as low (<600 MET-min/week), moderate (600–2999 MET-min/week), and high (≥3000 MET-min/week). Percentages are reported for the total sample and, where indicated, for valid IPAQ cases only.

IPAQ total physical activity showed a positively skewed distribution and non-negligible missingness, and several raw responses required protocol-based preprocessing, including truncation of extreme duration values and exclusion of short activity bouts; in addition, some vigorous and moderate activity responses showed inconsistencies between reported days and minutes. Consequently, all preregistered analyses involving IPAQ total physical activity were conducted only on participants with valid IPAQ total MET-min/week values.

4 **Supplementary individual-difference analyses**

**4.1 NEO-FFI correlations — Table S10**

Supplementary Table S10 reports preregistered Pearson correlations between the five NEO-FFI domains and all setting-specific change scores across the indoor, urban walk-and-talk, and nature walk-and-talk conditions. Across the 240 tested associations (5 personality traits × 48 change scores), 11 reached nominal significance at *p* < .05, but none remained significant after Bonferroni correction. The strongest nominal association was observed between Openness to Experience and Somatic Anxiety (STICSA) change in the nature walk-and-talk setting (*r* = -.211, *p* = .002, *p*<sub>Bonf</sub> = .463). Overall, the preregistered H3 analyses did not provide robust support for associations between personality traits and intervention-related change across settings.

Table S10: Pearson correlations between NEO-FFI personality traits and all preregistered setting-specific change scores

| **Trait** | **Outcome** | **Setting** | **r** | **p** | **pBonf** | **Significant after Bonferroni** |
| --- | --- | --- | --- | --- | --- | --- |
| Neuroticism | Systolic blood pressure | Indoor | .094 | .172 | 1.000 | No |
| Neuroticism | Systolic blood pressure | Urban walk-and-talk | .029 | .678 | 1.000 | No |
| Neuroticism | Systolic blood pressure | Nature walk-and-talk | .085 | .216 | 1.000 | No |
| Neuroticism | Diastolic blood pressure | Indoor | .013 | .845 | 1.000 | No |
| Neuroticism | Diastolic blood pressure | Urban walk-and-talk | .137 | .046 | 1.000 | No |
| Neuroticism | Diastolic blood pressure | Nature walk-and-talk | .014 | .836 | 1.000 | No |
| Neuroticism | Heart rate | Indoor | .026 | .703 | 1.000 | No |
| Neuroticism | Heart rate | Urban walk-and-talk | -.005 | .945 | 1.000 | No |
| Neuroticism | Heart rate | Nature walk-and-talk | .018 | .798 | 1.000 | No |
| Neuroticism | Positive Affect (PANAS) | Indoor | -.071 | .303 | 1.000 | No |
| Neuroticism | Positive Affect (PANAS) | Urban walk-and-talk | .056 | .419 | 1.000 | No |
| Neuroticism | Positive Affect (PANAS) | Nature walk-and-talk | -.025 | .722 | 1.000 | No |
| Neuroticism | Negative Affect (PANAS) | Indoor | .032 | .647 | 1.000 | No |
| Neuroticism | Negative Affect (PANAS) | Urban walk-and-talk | .102 | .140 | 1.000 | No |
| Neuroticism | Negative Affect (PANAS) | Nature walk-and-talk | .018 | .795 | 1.000 | No |
| Neuroticism | Vigor (POMS) | Indoor | -.056 | .414 | 1.000 | No |
| Neuroticism | Vigor (POMS) | Urban walk-and-talk | .152 | .027 | 1.000 | No |
| Neuroticism | Vigor (POMS) | Nature walk-and-talk | -.095 | .166 | 1.000 | No |
| Neuroticism | Anger (POMS) | Indoor | -.038 | .580 | 1.000 | No |
| Neuroticism | Anger (POMS) | Urban walk-and-talk | -.055 | .428 | 1.000 | No |
| Neuroticism | Anger (POMS) | Nature walk-and-talk | .109 | .113 | 1.000 | No |
| Neuroticism | Fatigue (POMS) | Indoor | .013 | .847 | 1.000 | No |
| Neuroticism | Fatigue (POMS) | Urban walk-and-talk | -.004 | .950 | 1.000 | No |
| Neuroticism | Fatigue (POMS) | Nature walk-and-talk | .021 | .763 | 1.000 | No |
| Neuroticism | Dejection (POMS) | Indoor | -.021 | .760 | 1.000 | No |
| Neuroticism | Dejection (POMS) | Urban walk-and-talk | .006 | .925 | 1.000 | No |
| Neuroticism | Dejection (POMS) | Nature walk-and-talk | .063 | .364 | 1.000 | No |
| Neuroticism | Positive Affect (ZIPERS) | Indoor | .049 | .476 | 1.000 | No |
| Neuroticism | Positive Affect (ZIPERS) | Urban walk-and-talk | .039 | .573 | 1.000 | No |
| Neuroticism | Positive Affect (ZIPERS) | Nature walk-and-talk | .012 | .858 | 1.000 | No |
| Neuroticism | Attention (ZIPERS) | Indoor | .142 | .039 | 1.000 | No |
| Neuroticism | Attention (ZIPERS) | Urban walk-and-talk | .103 | .135 | 1.000 | No |
| Neuroticism | Attention (ZIPERS) | Nature walk-and-talk | -.117 | .088 | 1.000 | No |
| Neuroticism | Fear (ZIPERS) | Indoor | -.056 | .415 | 1.000 | No |
| Neuroticism | Fear (ZIPERS) | Urban walk-and-talk | .051 | .460 | 1.000 | No |
| Neuroticism | Fear (ZIPERS) | Nature walk-and-talk | -.065 | .347 | 1.000 | No |
| Neuroticism | Sadness (ZIPERS) | Indoor | .084 | .222 | 1.000 | No |
| Neuroticism | Sadness (ZIPERS) | Urban walk-and-talk | .109 | .114 | 1.000 | No |
| Neuroticism | Sadness (ZIPERS) | Nature walk-and-talk | .087 | .209 | 1.000 | No |
| Neuroticism | Anger (ZIPERS) | Indoor | -.029 | .673 | 1.000 | No |
| Neuroticism | Anger (ZIPERS) | Urban walk-and-talk | -.078 | .259 | 1.000 | No |
| Neuroticism | Anger (ZIPERS) | Nature walk-and-talk | -.089 | .193 | 1.000 | No |
| Neuroticism | Cognitive Anxiety (STICSA) | Indoor | -.080 | .247 | 1.000 | No |
| Neuroticism | Cognitive Anxiety (STICSA) | Urban walk-and-talk | -.003 | .966 | 1.000 | No |
| Neuroticism | Cognitive Anxiety (STICSA) | Nature walk-and-talk | .005 | .938 | 1.000 | No |
| Neuroticism | Somatic Anxiety (STICSA) | Indoor | .023 | .738 | 1.000 | No |
| Neuroticism | Somatic Anxiety (STICSA) | Urban walk-and-talk | -.078 | .257 | 1.000 | No |
| Neuroticism | Somatic Anxiety (STICSA) | Nature walk-and-talk | -.044 | .527 | 1.000 | No |
| Extraversion | Systolic blood pressure | Indoor | -.006 | .928 | 1.000 | No |
| Extraversion | Systolic blood pressure | Urban walk-and-talk | -.013 | .855 | 1.000 | No |
| Extraversion | Systolic blood pressure | Nature walk-and-talk | -.069 | .319 | 1.000 | No |
| Extraversion | Diastolic blood pressure | Indoor | .003 | .960 | 1.000 | No |
| Extraversion | Diastolic blood pressure | Urban walk-and-talk | -.142 | .038 | 1.000 | No |
| Extraversion | Diastolic blood pressure | Nature walk-and-talk | -.033 | .630 | 1.000 | No |
| Extraversion | Heart rate | Indoor | -.017 | .810 | 1.000 | No |
| Extraversion | Heart rate | Urban walk-and-talk | .012 | .867 | 1.000 | No |
| Extraversion | Heart rate | Nature walk-and-talk | .052 | .453 | 1.000 | No |
| Extraversion | Positive Affect (PANAS) | Indoor | -.041 | .548 | 1.000 | No |
| Extraversion | Positive Affect (PANAS) | Urban walk-and-talk | -.004 | .952 | 1.000 | No |
| Extraversion | Positive Affect (PANAS) | Nature walk-and-talk | -.029 | .674 | 1.000 | No |
| Extraversion | Negative Affect (PANAS) | Indoor | .093 | .175 | 1.000 | No |
| Extraversion | Negative Affect (PANAS) | Urban walk-and-talk | -.065 | .348 | 1.000 | No |
| Extraversion | Negative Affect (PANAS) | Nature walk-and-talk | .062 | .368 | 1.000 | No |
| Extraversion | Vigor (POMS) | Indoor | .046 | .502 | 1.000 | No |
| Extraversion | Vigor (POMS) | Urban walk-and-talk | -.087 | .204 | 1.000 | No |
| Extraversion | Vigor (POMS) | Nature walk-and-talk | .044 | .522 | 1.000 | No |
| Extraversion | Anger (POMS) | Indoor | -.052 | .454 | 1.000 | No |
| Extraversion | Anger (POMS) | Urban walk-and-talk | .024 | .725 | 1.000 | No |
| Extraversion | Anger (POMS) | Nature walk-and-talk | .015 | .831 | 1.000 | No |
| Extraversion | Fatigue (POMS) | Indoor | .023 | .743 | 1.000 | No |
| Extraversion | Fatigue (POMS) | Urban walk-and-talk | .031 | .657 | 1.000 | No |
| Extraversion | Fatigue (POMS) | Nature walk-and-talk | .087 | .207 | 1.000 | No |
| Extraversion | Dejection (POMS) | Indoor | .028 | .680 | 1.000 | No |
| Extraversion | Dejection (POMS) | Urban walk-and-talk | .015 | .831 | 1.000 | No |
| Extraversion | Dejection (POMS) | Nature walk-and-talk | -.151 | .027 | 1.000 | No |
| Extraversion | Positive Affect (ZIPERS) | Indoor | -.011 | .870 | 1.000 | No |
| Extraversion | Positive Affect (ZIPERS) | Urban walk-and-talk | .063 | .362 | 1.000 | No |
| Extraversion | Positive Affect (ZIPERS) | Nature walk-and-talk | .034 | .621 | 1.000 | No |
| Extraversion | Attention (ZIPERS) | Indoor | -.023 | .737 | 1.000 | No |
| Extraversion | Attention (ZIPERS) | Urban walk-and-talk | -.064 | .355 | 1.000 | No |
| Extraversion | Attention (ZIPERS) | Nature walk-and-talk | .128 | .062 | 1.000 | No |
| Extraversion | Fear (ZIPERS) | Indoor | .080 | .247 | 1.000 | No |
| Extraversion | Fear (ZIPERS) | Urban walk-and-talk | -.006 | .930 | 1.000 | No |
| Extraversion | Fear (ZIPERS) | Nature walk-and-talk | .106 | .122 | 1.000 | No |
| Extraversion | Sadness (ZIPERS) | Indoor | -.031 | .649 | 1.000 | No |
| Extraversion | Sadness (ZIPERS) | Urban walk-and-talk | -.031 | .654 | 1.000 | No |
| Extraversion | Sadness (ZIPERS) | Nature walk-and-talk | .061 | .376 | 1.000 | No |
| Extraversion | Anger (ZIPERS) | Indoor | .070 | .306 | 1.000 | No |
| Extraversion | Anger (ZIPERS) | Urban walk-and-talk | -.050 | .468 | 1.000 | No |
| Extraversion | Anger (ZIPERS) | Nature walk-and-talk | -.025 | .713 | 1.000 | No |
| Extraversion | Cognitive Anxiety (STICSA) | Indoor | .132 | .055 | 1.000 | No |
| Extraversion | Cognitive Anxiety (STICSA) | Urban walk-and-talk | -.001 | .994 | 1.000 | No |
| Extraversion | Cognitive Anxiety (STICSA) | Nature walk-and-talk | .085 | .218 | 1.000 | No |
| Extraversion | Somatic Anxiety (STICSA) | Indoor | .012 | .857 | 1.000 | No |
| Extraversion | Somatic Anxiety (STICSA) | Urban walk-and-talk | .105 | .127 | 1.000 | No |
| Extraversion | Somatic Anxiety (STICSA) | Nature walk-and-talk | .013 | .848 | 1.000 | No |
| Openness to Experience | Systolic blood pressure | Indoor | .027 | .696 | 1.000 | No |
| Openness to Experience | Systolic blood pressure | Urban walk-and-talk | -.073 | .289 | 1.000 | No |
| Openness to Experience | Systolic blood pressure | Nature walk-and-talk | .001 | .993 | 1.000 | No |
| Openness to Experience | Diastolic blood pressure | Indoor | .039 | .573 | 1.000 | No |
| Openness to Experience | Diastolic blood pressure | Urban walk-and-talk | -.019 | .780 | 1.000 | No |
| Openness to Experience | Diastolic blood pressure | Nature walk-and-talk | -.019 | .788 | 1.000 | No |
| Openness to Experience | Heart rate | Indoor | .014 | .838 | 1.000 | No |
| Openness to Experience | Heart rate | Urban walk-and-talk | .005 | .938 | 1.000 | No |
| Openness to Experience | Heart rate | Nature walk-and-talk | -.044 | .521 | 1.000 | No |
| Openness to Experience | Positive Affect (PANAS) | Indoor | -.079 | .253 | 1.000 | No |
| Openness to Experience | Positive Affect (PANAS) | Urban walk-and-talk | .050 | .469 | 1.000 | No |
| Openness to Experience | Positive Affect (PANAS) | Nature walk-and-talk | .024 | .723 | 1.000 | No |
| Openness to Experience | Negative Affect (PANAS) | Indoor | .028 | .684 | 1.000 | No |
| Openness to Experience | Negative Affect (PANAS) | Urban walk-and-talk | -.032 | .643 | 1.000 | No |
| Openness to Experience | Negative Affect (PANAS) | Nature walk-and-talk | .015 | .822 | 1.000 | No |
| Openness to Experience | Vigor (POMS) | Indoor | -.011 | .876 | 1.000 | No |
| Openness to Experience | Vigor (POMS) | Urban walk-and-talk | -.113 | .099 | 1.000 | No |
| Openness to Experience | Vigor (POMS) | Nature walk-and-talk | .092 | .183 | 1.000 | No |
| Openness to Experience | Anger (POMS) | Indoor | .002 | .978 | 1.000 | No |
| Openness to Experience | Anger (POMS) | Urban walk-and-talk | .001 | .983 | 1.000 | No |
| Openness to Experience | Anger (POMS) | Nature walk-and-talk | -.102 | .139 | 1.000 | No |
| Openness to Experience | Fatigue (POMS) | Indoor | .031 | .655 | 1.000 | No |
| Openness to Experience | Fatigue (POMS) | Urban walk-and-talk | .059 | .388 | 1.000 | No |
| Openness to Experience | Fatigue (POMS) | Nature walk-and-talk | -.003 | .971 | 1.000 | No |
| Openness to Experience | Dejection (POMS) | Indoor | -.064 | .356 | 1.000 | No |
| Openness to Experience | Dejection (POMS) | Urban walk-and-talk | -.038 | .578 | 1.000 | No |
| Openness to Experience | Dejection (POMS) | Nature walk-and-talk | -.136 | .048 | 1.000 | No |
| Openness to Experience | Positive Affect (ZIPERS) | Indoor | -.021 | .765 | 1.000 | No |
| Openness to Experience | Positive Affect (ZIPERS) | Urban walk-and-talk | -.113 | .100 | 1.000 | No |
| Openness to Experience | Positive Affect (ZIPERS) | Nature walk-and-talk | .109 | .111 | 1.000 | No |
| Openness to Experience | Attention (ZIPERS) | Indoor | .013 | .851 | 1.000 | No |
| Openness to Experience | Attention (ZIPERS) | Urban walk-and-talk | -.133 | .053 | 1.000 | No |
| Openness to Experience | Attention (ZIPERS) | Nature walk-and-talk | .130 | .057 | 1.000 | No |
| Openness to Experience | Fear (ZIPERS) | Indoor | .033 | .627 | 1.000 | No |
| Openness to Experience | Fear (ZIPERS) | Urban walk-and-talk | .001 | .986 | 1.000 | No |
| Openness to Experience | Fear (ZIPERS) | Nature walk-and-talk | -.012 | .867 | 1.000 | No |
| Openness to Experience | Sadness (ZIPERS) | Indoor | -.132 | .055 | 1.000 | No |
| Openness to Experience | Sadness (ZIPERS) | Urban walk-and-talk | -.041 | .549 | 1.000 | No |
| Openness to Experience | Sadness (ZIPERS) | Nature walk-and-talk | -.041 | .548 | 1.000 | No |
| Openness to Experience | Anger (ZIPERS) | Indoor | -.004 | .952 | 1.000 | No |
| Openness to Experience | Anger (ZIPERS) | Urban walk-and-talk | -.096 | .162 | 1.000 | No |
| Openness to Experience | Anger (ZIPERS) | Nature walk-and-talk | -.042 | .544 | 1.000 | No |
| Openness to Experience | Cognitive Anxiety (STICSA) | Indoor | -.001 | .987 | 1.000 | No |
| Openness to Experience | Cognitive Anxiety (STICSA) | Urban walk-and-talk | .000 | 1.000 | 1.000 | No |
| Openness to Experience | Cognitive Anxiety (STICSA) | Nature walk-and-talk | -.053 | .439 | 1.000 | No |
| Openness to Experience | Somatic Anxiety (STICSA) | Indoor | .059 | .390 | 1.000 | No |
| Openness to Experience | Somatic Anxiety (STICSA) | Urban walk-and-talk | .018 | .797 | 1.000 | No |
| Openness to Experience | Somatic Anxiety (STICSA) | Nature walk-and-talk | -.211 | .002 | .463 | No |
| Agreeableness | Systolic blood pressure | Indoor | -.133 | .052 | 1.000 | No |
| Agreeableness | Systolic blood pressure | Urban walk-and-talk | -.022 | .750 | 1.000 | No |
| Agreeableness | Systolic blood pressure | Nature walk-and-talk | .051 | .462 | 1.000 | No |
| Agreeableness | Diastolic blood pressure | Indoor | .054 | .434 | 1.000 | No |
| Agreeableness | Diastolic blood pressure | Urban walk-and-talk | -.020 | .776 | 1.000 | No |
| Agreeableness | Diastolic blood pressure | Nature walk-and-talk | .010 | .889 | 1.000 | No |
| Agreeableness | Heart rate | Indoor | .040 | .557 | 1.000 | No |
| Agreeableness | Heart rate | Urban walk-and-talk | .034 | .625 | 1.000 | No |
| Agreeableness | Heart rate | Nature walk-and-talk | .005 | .939 | 1.000 | No |
| Agreeableness | Positive Affect (PANAS) | Indoor | -.080 | .248 | 1.000 | No |
| Agreeableness | Positive Affect (PANAS) | Urban walk-and-talk | .045 | .512 | 1.000 | No |
| Agreeableness | Positive Affect (PANAS) | Nature walk-and-talk | -.038 | .579 | 1.000 | No |
| Agreeableness | Negative Affect (PANAS) | Indoor | .120 | .079 | 1.000 | No |
| Agreeableness | Negative Affect (PANAS) | Urban walk-and-talk | .018 | .791 | 1.000 | No |
| Agreeableness | Negative Affect (PANAS) | Nature walk-and-talk | .083 | .229 | 1.000 | No |
| Agreeableness | Vigor (POMS) | Indoor | .069 | .320 | 1.000 | No |
| Agreeableness | Vigor (POMS) | Urban walk-and-talk | -.069 | .316 | 1.000 | No |
| Agreeableness | Vigor (POMS) | Nature walk-and-talk | .087 | .207 | 1.000 | No |
| Agreeableness | Anger (POMS) | Indoor | -.061 | .372 | 1.000 | No |
| Agreeableness | Anger (POMS) | Urban walk-and-talk | .060 | .386 | 1.000 | No |
| Agreeableness | Anger (POMS) | Nature walk-and-talk | .040 | .559 | 1.000 | No |
| Agreeableness | Fatigue (POMS) | Indoor | .021 | .763 | 1.000 | No |
| Agreeableness | Fatigue (POMS) | Urban walk-and-talk | .068 | .324 | 1.000 | No |
| Agreeableness | Fatigue (POMS) | Nature walk-and-talk | .114 | .096 | 1.000 | No |
| Agreeableness | Dejection (POMS) | Indoor | -.075 | .276 | 1.000 | No |
| Agreeableness | Dejection (POMS) | Urban walk-and-talk | -.037 | .588 | 1.000 | No |
| Agreeableness | Dejection (POMS) | Nature walk-and-talk | -.116 | .090 | 1.000 | No |
| Agreeableness | Positive Affect (ZIPERS) | Indoor | -.031 | .654 | 1.000 | No |
| Agreeableness | Positive Affect (ZIPERS) | Urban walk-and-talk | -.003 | .969 | 1.000 | No |
| Agreeableness | Positive Affect (ZIPERS) | Nature walk-and-talk | .119 | .084 | 1.000 | No |
| Agreeableness | Attention (ZIPERS) | Indoor | -.001 | .993 | 1.000 | No |
| Agreeableness | Attention (ZIPERS) | Urban walk-and-talk | -.086 | .214 | 1.000 | No |
| Agreeableness | Attention (ZIPERS) | Nature walk-and-talk | .154 | .024 | 1.000 | No |
| Agreeableness | Fear (ZIPERS) | Indoor | .124 | .070 | 1.000 | No |
| Agreeableness | Fear (ZIPERS) | Urban walk-and-talk | -.004 | .959 | 1.000 | No |
| Agreeableness | Fear (ZIPERS) | Nature walk-and-talk | .058 | .397 | 1.000 | No |
| Agreeableness | Sadness (ZIPERS) | Indoor | -.037 | .588 | 1.000 | No |
| Agreeableness | Sadness (ZIPERS) | Urban walk-and-talk | .008 | .903 | 1.000 | No |
| Agreeableness | Sadness (ZIPERS) | Nature walk-and-talk | .012 | .862 | 1.000 | No |
| Agreeableness | Anger (ZIPERS) | Indoor | .073 | .288 | 1.000 | No |
| Agreeableness | Anger (ZIPERS) | Urban walk-and-talk | .029 | .671 | 1.000 | No |
| Agreeableness | Anger (ZIPERS) | Nature walk-and-talk | -.049 | .479 | 1.000 | No |
| Agreeableness | Cognitive Anxiety (STICSA) | Indoor | .013 | .846 | 1.000 | No |
| Agreeableness | Cognitive Anxiety (STICSA) | Urban walk-and-talk | -.052 | .447 | 1.000 | No |
| Agreeableness | Cognitive Anxiety (STICSA) | Nature walk-and-talk | .003 | .965 | 1.000 | No |
| Agreeableness | Somatic Anxiety (STICSA) | Indoor | .023 | .739 | 1.000 | No |
| Agreeableness | Somatic Anxiety (STICSA) | Urban walk-and-talk | .093 | .177 | 1.000 | No |
| Agreeableness | Somatic Anxiety (STICSA) | Nature walk-and-talk | -.057 | .404 | 1.000 | No |
| Conscientiousness | Systolic blood pressure | Indoor | .000 | .996 | 1.000 | No |
| Conscientiousness | Systolic blood pressure | Urban walk-and-talk | .095 | .166 | 1.000 | No |
| Conscientiousness | Systolic blood pressure | Nature walk-and-talk | .041 | .556 | 1.000 | No |
| Conscientiousness | Diastolic blood pressure | Indoor | -.034 | .618 | 1.000 | No |
| Conscientiousness | Diastolic blood pressure | Urban walk-and-talk | -.017 | .802 | 1.000 | No |
| Conscientiousness | Diastolic blood pressure | Nature walk-and-talk | .026 | .710 | 1.000 | No |
| Conscientiousness | Heart rate | Indoor | -.040 | .558 | 1.000 | No |
| Conscientiousness | Heart rate | Urban walk-and-talk | -.008 | .906 | 1.000 | No |
| Conscientiousness | Heart rate | Nature walk-and-talk | .006 | .927 | 1.000 | No |
| Conscientiousness | Positive Affect (PANAS) | Indoor | .074 | .283 | 1.000 | No |
| Conscientiousness | Positive Affect (PANAS) | Urban walk-and-talk | .036 | .600 | 1.000 | No |
| Conscientiousness | Positive Affect (PANAS) | Nature walk-and-talk | .045 | .510 | 1.000 | No |
| Conscientiousness | Negative Affect (PANAS) | Indoor | -.082 | .234 | 1.000 | No |
| Conscientiousness | Negative Affect (PANAS) | Urban walk-and-talk | -.057 | .412 | 1.000 | No |
| Conscientiousness | Negative Affect (PANAS) | Nature walk-and-talk | -.030 | .666 | 1.000 | No |
| Conscientiousness | Vigor (POMS) | Indoor | -.039 | .571 | 1.000 | No |
| Conscientiousness | Vigor (POMS) | Urban walk-and-talk | .072 | .295 | 1.000 | No |
| Conscientiousness | Vigor (POMS) | Nature walk-and-talk | -.022 | .748 | 1.000 | No |
| Conscientiousness | Anger (POMS) | Indoor | .105 | .126 | 1.000 | No |
| Conscientiousness | Anger (POMS) | Urban walk-and-talk | -.024 | .728 | 1.000 | No |
| Conscientiousness | Anger (POMS) | Nature walk-and-talk | .052 | .452 | 1.000 | No |
| Conscientiousness | Fatigue (POMS) | Indoor | .015 | .831 | 1.000 | No |
| Conscientiousness | Fatigue (POMS) | Urban walk-and-talk | -.100 | .145 | 1.000 | No |
| Conscientiousness | Fatigue (POMS) | Nature walk-and-talk | -.009 | .897 | 1.000 | No |
| Conscientiousness | Dejection (POMS) | Indoor | .076 | .267 | 1.000 | No |
| Conscientiousness | Dejection (POMS) | Urban walk-and-talk | .030 | .662 | 1.000 | No |
| Conscientiousness | Dejection (POMS) | Nature walk-and-talk | .060 | .382 | 1.000 | No |
| Conscientiousness | Positive Affect (ZIPERS) | Indoor | -.023 | .737 | 1.000 | No |
| Conscientiousness | Positive Affect (ZIPERS) | Urban walk-and-talk | .165 | .016 | 1.000 | No |
| Conscientiousness | Positive Affect (ZIPERS) | Nature walk-and-talk | -.096 | .165 | 1.000 | No |
| Conscientiousness | Attention (ZIPERS) | Indoor | -.066 | .335 | 1.000 | No |
| Conscientiousness | Attention (ZIPERS) | Urban walk-and-talk | .062 | .365 | 1.000 | No |
| Conscientiousness | Attention (ZIPERS) | Nature walk-and-talk | -.052 | .450 | 1.000 | No |
| Conscientiousness | Fear (ZIPERS) | Indoor | .027 | .690 | 1.000 | No |
| Conscientiousness | Fear (ZIPERS) | Urban walk-and-talk | .024 | .732 | 1.000 | No |
| Conscientiousness | Fear (ZIPERS) | Nature walk-and-talk | .007 | .917 | 1.000 | No |
| Conscientiousness | Sadness (ZIPERS) | Indoor | .078 | .256 | 1.000 | No |
| Conscientiousness | Sadness (ZIPERS) | Urban walk-and-talk | -.143 | .037 | 1.000 | No |
| Conscientiousness | Sadness (ZIPERS) | Nature walk-and-talk | .025 | .717 | 1.000 | No |
| Conscientiousness | Anger (ZIPERS) | Indoor | -.106 | .124 | 1.000 | No |
| Conscientiousness | Anger (ZIPERS) | Urban walk-and-talk | .033 | .636 | 1.000 | No |
| Conscientiousness | Anger (ZIPERS) | Nature walk-and-talk | .145 | .034 | 1.000 | No |
| Conscientiousness | Cognitive Anxiety (STICSA) | Indoor | -.043 | .529 | 1.000 | No |
| Conscientiousness | Cognitive Anxiety (STICSA) | Urban walk-and-talk | -.075 | .274 | 1.000 | No |
| Conscientiousness | Cognitive Anxiety (STICSA) | Nature walk-and-talk | .016 | .812 | 1.000 | No |
| Conscientiousness | Somatic Anxiety (STICSA) | Indoor | .053 | .438 | 1.000 | No |
| Conscientiousness | Somatic Anxiety (STICSA) | Urban walk-and-talk | -.036 | .597 | 1.000 | No |
| Conscientiousness | Somatic Anxiety (STICSA) | Nature walk-and-talk | .132 | .055 | 1.000 | No |

**Note.** N = 213. NEO-FFI = NEO Five-Factor Inventory; PANAS = Positive and Negative Affect Schedule; POMS = Profile of Mood States; ZIPERS = Zuckerman Inventory of Personal Reactions; STICSA = State-Trait Inventory for Cognitive and Somatic Anxiety; pBonf = Bonferroni-corrected p value. Pearson correlation analyses were conducted between the five NEO-FFI domains and all preregistered setting-specific change scores. Change scores were calculated as post-intervention minus pre-intervention values. Bonferroni-corrected p values reflect correction across the 240 preregistered H3 tests. No association remained statistically significant after Bonferroni correction.

**4.2 Environmental Identity correlations — Table S11**

**Environmental Identity and setting-specific change scores.** Supplementary Table S11 reports preregistered Pearson correlations between Environmental Identity and all setting-specific change scores across the indoor, urban walk-and-talk, and nature walk-and-talk conditions. Across the 48 tested associations (1 predictor × 48 change scores), 2 reached nominal significance at *p* < .05, but none remained significant after Bonferroni correction. The strongest nominal association was observed between Environmental Identity and Somatic Anxiety (STICSA) change in the nature walk-and-talk setting (*r* = -.170, *p* = .013, *p*<sub>Bonf</sub> = .613). Overall, the preregistered H4 analyses did not provide robust support for associations between Environmental Identity and intervention-related change across settings.

Table S11. Pearson correlations between Environmental Identity and all preregistered setting-specific change scores

| **Outcome** | **Setting** | **r** | **p** | **pBonf** | **Significant after Bonferroni** |
| --- | --- | --- | --- | --- | --- |
| Positive Affect (PANAS) | Indoor | -.027 | .697 | 1.000 | No |
| Positive Affect (PANAS) | Urban walk-and-talk | -.030 | .660 | 1.000 | No |
| Positive Affect (PANAS) | Nature walk-and-talk | .052 | .449 | 1.000 | No |
| Negative Affect (PANAS) | Indoor | .025 | .714 | 1.000 | No |
| Negative Affect (PANAS) | Urban walk-and-talk | -.115 | .095 | 1.000 | No |
| Negative Affect (PANAS) | Nature walk-and-talk | .015 | .828 | 1.000 | No |
| Vigor (POMS) | Indoor | .073 | .286 | 1.000 | No |
| Vigor (POMS) | Urban walk-and-talk | .100 | .147 | 1.000 | No |
| Vigor (POMS) | Nature walk-and-talk | -.025 | .719 | 1.000 | No |
| Anger (POMS) | Indoor | -.056 | .419 | 1.000 | No |
| Anger (POMS) | Urban walk-and-talk | .029 | .673 | 1.000 | No |
| Anger (POMS) | Nature walk-and-talk | .038 | .578 | 1.000 | No |
| Fatigue (POMS) | Indoor | -.040 | .563 | 1.000 | No |
| Fatigue (POMS) | Urban walk-and-talk | -.103 | .133 | 1.000 | No |
| Fatigue (POMS) | Nature walk-and-talk | -.004 | .954 | 1.000 | No |
| Dejection (POMS) | Indoor | -.022 | .750 | 1.000 | No |
| Dejection (POMS) | Urban walk-and-talk | -.075 | .279 | 1.000 | No |
| Dejection (POMS) | Nature walk-and-talk | .127 | .064 | 1.000 | No |
| Positive Affect (ZIPERS) | Indoor | -.011 | .870 | 1.000 | No |
| Positive Affect (ZIPERS) | Urban walk-and-talk | .033 | .631 | 1.000 | No |
| Positive Affect (ZIPERS) | Nature walk-and-talk | .081 | .241 | 1.000 | No |
| Attention (ZIPERS) | Indoor | -.082 | .233 | 1.000 | No |
| Attention (ZIPERS) | Urban walk-and-talk | .094 | .169 | 1.000 | No |
| Attention (ZIPERS) | Nature walk-and-talk | .097 | .159 | 1.000 | No |
| Fear (ZIPERS) | Indoor | -.032 | .644 | 1.000 | No |
| Fear (ZIPERS) | Urban walk-and-talk | -.092 | .182 | 1.000 | No |
| Fear (ZIPERS) | Nature walk-and-talk | -.006 | .931 | 1.000 | No |
| Sadness (ZIPERS) | Indoor | .025 | .715 | 1.000 | No |
| Sadness (ZIPERS) | Urban walk-and-talk | .041 | .550 | 1.000 | No |
| Sadness (ZIPERS) | Nature walk-and-talk | -.077 | .264 | 1.000 | No |
| Anger (ZIPERS) | Indoor | -.102 | .136 | 1.000 | No |
| Anger (ZIPERS) | Urban walk-and-talk | .058 | .396 | 1.000 | No |
| Anger (ZIPERS) | Nature walk-and-talk | .035 | .611 | 1.000 | No |
| Cognitive Anxiety (STICSA) | Indoor | -.035 | .612 | 1.000 | No |
| Cognitive Anxiety (STICSA) | Urban walk-and-talk | .006 | .926 | 1.000 | No |
| Cognitive Anxiety (STICSA) | Nature walk-and-talk | -.139 | .042 | 1.000 | No |
| Somatic Anxiety (STICSA) | Indoor | -.021 | .765 | 1.000 | No |
| Somatic Anxiety (STICSA) | Urban walk-and-talk | -.086 | .211 | 1.000 | No |
| Somatic Anxiety (STICSA) | Nature walk-and-talk | -.170 | .013 | .613 | No |
| Systolic blood pressure | Indoor | -.054 | .430 | 1.000 | No |
| Systolic blood pressure | Urban walk-and-talk | -.110 | .110 | 1.000 | No |
| Systolic blood pressure | Nature walk-and-talk | -.035 | .612 | 1.000 | No |
| Diastolic blood pressure | Indoor | .007 | .915 | 1.000 | No |
| Diastolic blood pressure | Urban walk-and-talk | .031 | .652 | 1.000 | No |
| Diastolic blood pressure | Nature walk-and-talk | .068 | .326 | 1.000 | No |
| Heart rate | Indoor | .063 | .361 | 1.000 | No |
| Heart rate | Urban walk-and-talk | .076 | .268 | 1.000 | No |
| Heart rate | Nature walk-and-talk | -.089 | .194 | 1.000 | No |

**Note.** N = 213. PANAS = Positive and Negative Affect Schedule; POMS = Profile of Mood States; ZIPERS = Zuckerman Inventory of Personal Reactions; STICSA = State-Trait Inventory for Cognitive and Somatic Anxiety; pBonf = Bonferroni-corrected p value. Pearson correlation analyses were conducted between Environmental Identity and all preregistered setting-specific change scores. The Environmental Identity total score was computed as the mean of 14 items. Change scores were calculated as post-intervention minus pre-intervention values. Bonferroni-corrected p values reflect correction across the 48 preregistered H4 tests. No association remained statistically significant after Bonferroni correction.

**4.3 IPAQ correlations — Table S12**

**Physical activity and setting-specific change scores.** Supplementary Table S12 reports preregistered Pearson correlations between physical activity and all setting-specific change scores across the indoor, urban walk-and-talk, and nature walk-and-talk conditions. For these analyses, physical activity was operationalized as total IPAQ physical activity expressed in MET-min/week, because Pearson correlation requires a continuous indicator. Across the 48 tested associations (1 predictor × 48 change scores), 1 reached nominal significance at p < .05, but none remained significant after Bonferroni correction. The strongest nominal association was observed between total physical activity and systolic blood pressure change in the indoor setting (r = .156, p = .034, p<sub>Bonf</sub> = 1.000). Overall, the preregistered H5 analyses did not provide robust support for associations between physical activity and intervention-related change across settings.

Table S12. Pearson correlations between total physical activity (IPAQ total MET-min/week) and all preregistered setting-specific change scores

| **Outcome** | **Setting** | **r** | **p** | **pBonf** | **Significant after Bonferroni** |
| --- | --- | --- | --- | --- | --- |
| Systolic blood pressure | Indoor | .156 | .034 | 1.000 | No |
| Diastolic blood pressure | Indoor | .077 | .300 | 1.000 | No |
| Heart rate | Indoor | -.095 | .199 | 1.000 | No |
| Systolic blood pressure | Urban walk-and-talk | -.096 | .193 | 1.000 | No |
| Diastolic blood pressure | Urban walk-and-talk | -.069 | .352 | 1.000 | No |
| Heart rate | Urban walk-and-talk | .076 | .306 | 1.000 | No |
| Systolic blood pressure | Nature walk-and-talk | -.054 | .465 | 1.000 | No |
| Diastolic blood pressure | Nature walk-and-talk | .068 | .356 | 1.000 | No |
| Heart rate | Nature walk-and-talk | -.034 | .644 | 1.000 | No |
| Positive Affect (PANAS) | Indoor | -.101 | .170 | 1.000 | No |
| Negative Affect (PANAS) | Indoor | -.029 | .699 | 1.000 | No |
| Vigor (POMS) | Indoor | .023 | .758 | 1.000 | No |
| Anger (POMS) | Indoor | .066 | .373 | 1.000 | No |
| Fatigue (POMS) | Indoor | -.077 | .296 | 1.000 | No |
| Dejection (POMS) | Indoor | -.013 | .859 | 1.000 | No |
| Positive Affect (ZIPERS) | Indoor | .071 | .333 | 1.000 | No |
| Attention (ZIPERS) | Indoor | .112 | .130 | 1.000 | No |
| Fear (ZIPERS) | Indoor | -.054 | .468 | 1.000 | No |
| Sadness (ZIPERS) | Indoor | -.033 | .660 | 1.000 | No |
| Anger (ZIPERS) | Indoor | .101 | .172 | 1.000 | No |
| Cognitive Anxiety (STICSA) | Indoor | -.038 | .611 | 1.000 | No |
| Somatic Anxiety (STICSA) | Indoor | -.006 | .936 | 1.000 | No |
| Positive Affect (PANAS) | Urban walk-and-talk | .055 | .459 | 1.000 | No |
| Negative Affect (PANAS) | Urban walk-and-talk | -.054 | .462 | 1.000 | No |
| Vigor (POMS) | Urban walk-and-talk | -.089 | .229 | 1.000 | No |
| Anger (POMS) | Urban walk-and-talk | -.096 | .192 | 1.000 | No |
| Fatigue (POMS) | Urban walk-and-talk | -.073 | .325 | 1.000 | No |
| Dejection (POMS) | Urban walk-and-talk | .072 | .330 | 1.000 | No |
| Positive Affect (ZIPERS) | Urban walk-and-talk | .031 | .677 | 1.000 | No |
| Attention (ZIPERS) | Urban walk-and-talk | .034 | .649 | 1.000 | No |
| Fear (ZIPERS) | Urban walk-and-talk | .017 | .820 | 1.000 | No |
| Sadness (ZIPERS) | Urban walk-and-talk | -.014 | .854 | 1.000 | No |
| Anger (ZIPERS) | Urban walk-and-talk | -.024 | .745 | 1.000 | No |
| Cognitive Anxiety (STICSA) | Urban walk-and-talk | -.029 | .692 | 1.000 | No |
| Somatic Anxiety (STICSA) | Urban walk-and-talk | .040 | .587 | 1.000 | No |
| Positive Affect (PANAS) | Nature walk-and-talk | .044 | .553 | 1.000 | No |
| Negative Affect (PANAS) | Nature walk-and-talk | .015 | .836 | 1.000 | No |
| Vigor (POMS) | Nature walk-and-talk | -.051 | .495 | 1.000 | No |
| Anger (POMS) | Nature walk-and-talk | .096 | .195 | 1.000 | No |
| Fatigue (POMS) | Nature walk-and-talk | .073 | .322 | 1.000 | No |
| Dejection (POMS) | Nature walk-and-talk | -.099 | .181 | 1.000 | No |
| Positive Affect (ZIPERS) | Nature walk-and-talk | .037 | .619 | 1.000 | No |
| Attention (ZIPERS) | Nature walk-and-talk | .096 | .194 | 1.000 | No |
| Fear (ZIPERS) | Nature walk-and-talk | .006 | .932 | 1.000 | No |
| Sadness (ZIPERS) | Nature walk-and-talk | .056 | .446 | 1.000 | No |
| Anger (ZIPERS) | Nature walk-and-talk | .020 | .791 | 1.000 | No |
| Cognitive Anxiety (STICSA) | Nature walk-and-talk | -.019 | .800 | 1.000 | No |
| Somatic Anxiety (STICSA) | Nature walk-and-talk | -.066 | .374 | 1.000 | No |

**Note.** N valid = 185, missing = 28. IPAQ = International Physical Activity Questionnaire; MET = metabolic equivalent of task; PANAS = Positive and Negative Affect Schedule; POMS = Profile of Mood States; ZIPERS = Zuckerman Inventory of Personal Reactions; STICSA = State-Trait Inventory for Cognitive and Somatic Anxiety; pBonf = Bonferroni-corrected p value. Pearson correlation analyses were conducted between total physical activity (IPAQ total MET-min/week) and all preregistered setting-specific change scores. Change scores were calculated as post-intervention minus pre-intervention values. Bonferroni-corrected p values reflect correction across the 48 preregistered H5 tests. No association remained statistically significant after Bonferroni correction.

**4.4 Exploratory mixed ANOVAs by IPAQ activity level — Table S13**

**Exploratory analysis of physical activity level.** As an exploratory follow-up to the preregistered H5 analyses, mixed ANOVAs were conducted with setting (indoor, urban walk-and-talk, nature walk-and-talk) as a within-subject factor and IPAQ activity level (low, moderate, high) as a between-subject factor. The analyses were based on participants with a valid IPAQ classification (*N* = 185; low = 33, moderate = 96, high = 56). No robust main effects of physical activity level or setting × physical activity level interactions were observed across outcomes. Only the interaction for POMS Anger reached nominal significance, F(4, 364) = 2.41, p = .049, ηp² = .026; however, this effect should be interpreted cautiously given the exploratory nature of the analysis and the number of tests conducted.

Table S13. Exploratory mixed ANOVA results for setting and IPAQ activity level across preregistered change scores

| **Outcome** | **IPAQ activity level,**  **F(2, 182)** | **IPAQ activity level, p** | **IPAQ activity level, ηp²** | **Setting × IPAQ activity level, F(4, 364)** | **Setting × IPAQ activity level, p** | **Setting × IPAQ activity level, ηp²** |
| --- | --- | --- | --- | --- | --- | --- |
| Systolic blood pressure | 0.40 | .671 | .004 | 1.29 | .275 | .014 |
| Diastolic blood pressure | 2.23 | .111 | .024 | 1.43 | .225 | .015 |
| Heart rate | 1.96 | .144 | .021 | 1.77 | .134 | .019 |
| Positive Affect (PANAS) | 2.59 | .077 | .028 | 0.82 | .514 | .009 |
| Negative Affect (PANAS) | 0.92 | .400 | .010 | 1.94 | .104 | .021 |
| Vigor (POMS) | 0.47 | .628 | .005 | 0.33 | .858 | .004 |
| Anger (POMS) | 0.33 | .720 | .004 | 2.41 | .049 | .026 |
| Fatigue (POMS) | 0.78 | .460 | .008 | 0.74 | .568 | .008 |
| Dejection (POMS) | 0.83 | .437 | .009 | 2.15 | .074 | .023 |
| Positive Affect (ZIPERS) | 0.16 | .853 | .002 | 0.72 | .577 | .008 |
| Attention (ZIPERS) | 0.64 | .529 | .007 | 1.58 | .179 | .017 |
| Fear (ZIPERS) | 0.51 | .603 | .006 | 0.54 | .709 | .006 |
| Sadness (ZIPERS) | 0.89 | .413 | .010 | 1.88 | .114 | .020 |
| Anger (ZIPERS) | 2.18 | .115 | .023 | 0.90 | .466 | .010 |
| Cognitive Anxiety (STICSA) | 0.79 | .455 | .009 | 0.85 | .492 | .009 |
| Somatic Anxiety (STICSA) | 0.88 | .416 | .010 | 0.15 | .961 | .002 |

**Note.** IPAQ = International Physical Activity Questionnaire; PANAS = Positive and Negative Affect Schedule; POMS = Profile of Mood States; ZIPERS = Zuckerman Inventory of Personal Reactions; STICSA = State-Trait Inventory for Cognitive and Somatic Anxiety; ηp² = partial eta squared. Exploratory mixed ANOVAs were conducted with setting as a within-subject factor and IPAQ activity level (low, moderate, high) as a between-subject factor. Reported values for the main effect of IPAQ level are based on F(2, 182), and reported values for the setting × IPAQ level interaction are based on F(4, 364). Change scores were calculated as post-intervention minus pre-intervention values. Analyses were based on participants with a valid IPAQ classification only (N = 185; low = 33, moderate = 96, high = 56). This analysis was exploratory and was not part of the preregistered primary testing strategy.
