# Supplementary material for: From indoor psychotherapy and counseling to walk-and-talk: psychological and physiological responses across a fixed sequence of indoor, urban, and nature-based sessions
Source: Front Psychol. 2026 Aug 13;17:1880259. doi: 10.3389/fpsyg.2026.1880259 (PMC13518116; doi:10.3389/fpsyg.2026.1880259)
Supplement: Supplementary file 2 [file Supplementary_file_2.DOCX]

Supplementary Material 2

# Paired TOST analyses for setting contrasts

Equivalence and minimum-effect analyses were conducted as supplementary analyses to clarify whether selected setting contrasts reflected practical similarity or exceeded prespecified smallest effect sizes of interest. Because jamovi does not provide omnibus equivalence or minimum-effect testing for repeated-measures ANOVA, these analyses were implemented at the level of the three planned within-subject setting contrasts: indoor versus urban walk-and-talk, indoor versus nature walk-and-talk, and urban versus nature walk-and-talk. For outcomes without a robust omnibus setting-related difference, paired TOST procedures were used primarily to test equivalence across setting contrasts. For outcomes with a robust omnibus setting-related difference, paired TOST procedures were used to test minimum effects for contrasts showing a meaningful pairwise pattern and equivalence for contrasts that appeared practically similar. These analyses should therefore be understood as contrast-level supplementary analyses rather than as a direct omnibus implementation of equivalence testing. Raw bounds were defined separately by outcome family as the largest differences considered practically negligible in the original metric of each measure: ±0.20 for mean-based PANAS, POMS, and multi-item ZIPERS outcomes; ±0.50 for ZIPERS Sadness; ±0.10 for STICSA outcomes; ±2.0 mmHg for systolic blood pressure; ±1.5 mmHg for diastolic blood pressure; and ±2.0 bpm for heart rate. Mean differences were calculated as the second setting minus the first setting in each contrast. For equivalence tests, statistical equivalence was concluded only when both one-sided TOST p values were significant. For minimum-effect tests, conclusions were based on the one-sided test corresponding to the expected direction of the contrast.

**Table S14. Paired TOST results for the three setting contrasts across H1 and H2 outcomes**

| **Outcome** | **Contrast** | **Test type** | **Raw bound** | **NHST t(df)** | **NHSTp** | **TOST Lower t** | **TOST Lower p** | **TOST Upper t** | **TOST Upper p** | **Mean difference (second-first)** | **90% CI lower** | **90% CI upper** | **Conclusion** |
| --- | --- | --- | --- | --- | --- | --- | --- | --- | --- | --- | --- | --- | --- |
| PANAS  Positive Affect | Indoor vs Urban | EQU | ±0.20 | 2.535  (212) | .012 | 6.753 | 1.000 | -1.683 | .953 | .120 | .042 | .199 | Different, not equivalent |
| PANAS  Positive Affect | Indoor vs Nature | EQU | ±0.20 | 1.021  (212) | .308 | 7.711 | <.001 | -5.670 | <.001 | .031 | -.019 | .080 | Equivalent |
| PANAS  Positive Affect | Urban vs Nature | EQU | ±0.20 | -1.780  (212) | .076 | 2.190 | .015 | -5.751 | <.001 | -.090 | -.173 | -.006 | Equivalent |
| PANAS  Negative Affect | Indoor vs Urban | MET | ±0.20 | 3.013  (212) | .003 | 6.304 | 1.000 | -.278 | .609 | .183 | .083 | .284 | Different from zero, but does not exceed SESOI |
| PANAS  Negative Affect | Indoor vs Nature | EQU | ±0.20 | -.070  (212) | .944 | 9.868 | <.001 | -10.008 | <.001 | -.001 | -.035 | .032 | Equivalent |
| PANAS  Negative Affect | Urban vs Nature | MET | ±0.20 | -2.992  (212) | .003 | .251 | .599 | -6.236 | 1.000 | -.185 | -.286 | -.083 | Different from zero, but does not exceed SESOI |
| POMS  Vigor | Indoor vs Urban | EQU | ±0.20 | 1.551  (212) | .122 | 4.696 | <.001 | -1.595 | .560 | .099 | -.006 | .204 | Inconclusive |
| POMS  Vigor | Indoor vs Nature | MET | ±0.20 | 8.055  (212) | <.001 | 9.875 | 1.000 | 6.235 | <.001 | .885 | .703 | 1.066 | Exceeds SESOI |
| POMS  Vigor | Urban vs Nature | MET | ±0.20 | 7.761  (212) | <.001 | 9.734 | 1.000 | 5.787 | <.001 | .786 | .619 | .954 | Exceeds SESOI |
| POMS  Anger | Indoor vs Urban | EQU | ±0.20 | -1.140  (212) | .256 | 3.275 | <.001 | -5.555 | <.001 | -.052 | -.126 | .023 | Equivalent |
| POMS  Anger | Indoor vs Nature | EQU | ±0.20 | -1.352  (212) | .178 | 3.656 | <.001 | -6.361 | <.001 | -.054 | -.120 | .012 | Equivalent |
| POMS  Anger | Urban vs Nature | EQU | ±0.20 | -.047  (212) | .962 | 3.993 | <.001 | -4.088 | <.001 | -.002 | -.084 | .079 | Equivalent |
| POMS  Fatigue | Indoor vs Urban | EQU | ±0.20 | -.169  (212) | .866 | 2.718 | .004 | -3.057 | .001 | -.012 | -.126 | .103 | Equivalent |
| POMS  Fatigue | Indoor vs Nature | EQU | ±0.20 | -.269  (212) | .788 | 2.784 | .003 | -3.321 | <.001 | -.018 | -.126 | .091 | Equivalent |
| POMS  Fatigue | Urban vs Nature | EQU | ±0.20 | -.082  (212) | .935 | 2.697 | .004 | -2.860 | .002 | -.006 | -.167 | .156 | Equivalent |
| POMS  Dejection | Indoor vs Urban | EQU | ±0.20 | -.139  (212) | .890 | 3.801 | <.001 | -4.079 | <.001 | -.007 | -.091 | .077 | Equivalent |
| POMS  Dejection | Indoor vs Nature | MET | ±0.20 | -3.708  (212) | <.001 | -.501 | .309 | -6.916 | 1.000 | -.231 | -.334 | -.128 | Different from zero, but does not exceed SESOI |
| POMS  Dejection | Urban vs Nature | MET | ±0.20 | -3.639  (212) | <.001 | -.392 | .348 | -6.886 | 1.000 | -.224 | -.326 | -.122 | Different from zero, but does not exceed SESOI |
| ZIPERS  Positive Affect | Indoor vs Urban | EQU | ±0.20 | .679  (212) | .498 | 3.983 | <.001 | -2.625 | .005 | .041 | -.059 | .141 | Equivalent |
| ZIPERS  Positive Affect | Indoor vs Nature | EQU | ±0.20 | 1.465  (212) | .145 | 4.706 | <.001 | -1.777 | .039 | .090 | -.012 | .192 | Equivalent |
| ZIPERS  Positive Affect | Urban vs Nature | EQU | ±0.20 | 1.076  (212) | .283 | 5.443 | <.001 | -3.290 | <.001 | .049 | -.026 | .125 | Equivalent |
| ZIPERS  Attention | Indoor vs Urban | EQU | ±0.20 | 2.049  (212) | .042 | 4.408 | <.001 | -.310 | .378 | .174 | .034 | .314 | Different, not equivalent |
| ZIPERS  Attention | Indoor vs Nature | EQU | ±0.20 | .513  (212) | .609 | 2.697 | .004 | -1.672 | .048 | .047 | -.104 | .198 | Equivalent |
| ZIPERS  Attention | Urban vs Nature | EQU | ±0.20 | -1.253  (212) | .212 | .724 | .235 | -3.230 | <.001 | -.127 | -.294 | .040 | Inconclusive |
| ZIPERS  Fear | Indoor vs Urban | EQU | ±0.20 | .086  (212) | .931 | 1.753 | .041 | -1.926 | .028 | .009 | -.189 | .170 | Equivalent |
| ZIPERS  Fear | Indoor vs Nature | EQU | ±0.20 | -1.160  (212) | .247 | 1.994 | .024 | -4.313 | <.001 | -.074 | -.178 | .031 | Equivalent |
| ZIPERS  Fear | Urban vs Nature | EQU | ±0.20 | -.696  (212) | .487 | 1.473 | .071 | -2.865 | .002 | -.064 | -.216 | .088 | Inconclusive |
| ZIPERS  Sadness | Indoor vs Urban | EQU | ±0.50 | .685  (212) | .494 | 5.545 | <.001 | -4.176 | <.001 | .070 | -.100 | .240 | Equivalent |
| ZIPERS  Sadness | Indoor vs Nature | EQU | ±0.50 | 1.192  (212) | .235 | 6.268 | <.001 | -3.885 | <.001 | .117 | -.045 | .280 | Equivalent |
| ZIPERS  Sadness | Urban vs Nature | EQU | ±0.50 | .736  (212) | .462 | 8.579 | <.001 | -7.106 | <.001 | .047 | -.058 | .152 | Equivalent |
| ZIPERS  Anger | Indoor vs Urban | EQU | ±0.20 | -.367  (212) | .714 | 2.478 | .007 | -3.213 | <.001 | -.026 | -.142 | .090 | Equivalent |
| ZIPERS  Anger | Indoor vs Nature | EQU | ±0.20 | .036  (212) | .971 | 3.130 | <.001 | -3.057 | .001 | .002 | -.104 | .109 | Equivalent |
| ZIPERS  Anger | Urban vs Nature | EQU | ±0.20 | .567  (212) | .571 | 4.595 | <.001 | -3.461 | <.001 | .028 | -.054 | .110 | Equivalent |
| STICSA  Cognitive Anxiety | Indoor vs Urban | EQU | ±0.10 | -.769  (212) | .443 | 2.091 | .019 | -3.629 | <.001 | -.027 | -.085 | .031 | Equivalent |
| STICSA  Cognitive Anxiety | Indoor vs Nature | EQU | ±0.10 | -.278  (212) | .781 | 2.686 | .004 | -3.243 | <.001 | -.009 | -.065 | .046 | Equivalent |
| STICSA  Cognitive Anxiety | Urban vs Nature | EQU | ±0.10 | .458  (212) | .647 | 3.079 | .001 | -2.162 | .016 | .017 | -.046 | .081 | Equivalent |
| STICSA  Somatic Anxiety | Indoor vs Urban | EQU | ±0.10 | 1.503  (212) | .134 | 4.671 | <.001 | -1.666 | .049 | .047 | -.005 | .100 | Equivalent |
| STICSA  Somatic Anxiety | Indoor vs Nature | EQU | ±0.10 | 1.943  (212) | .053 | 6.000 | <.001 | -2.114 | .018 | .048 | .007 | .089 | Equivalent |
| STICSA  Somatic Anxiety | Urban vs Nature | EQU | ±0.10 | .017  (212) | .987 | 3.608 | <.001 | -3.575 | <.001 | .000 | -.046 | .046 | Equivalent |
| Systolic blood pressure | Indoor vs Urban | MET | ±2.0 mmHg | -4.674  (212) | <.001 | -2.695 | .004 | -6.654 | 1.000 | -4.722 | -6.391 | -3.052 | Exceeds SESOI |
| Systolic blood pressure | Indoor vs Nature | MET | ±2.0 mmHg | -3.765  (212) | <.001 | -1.994 | .024 | -5.536 | 1.000 | -4.252 | -6.118 | -2.387 | Exceeds SESOI |
| Systolic blood pressure | Urban vs Nature | EQU | ±2.0 mmHg | .890  (212) | .375 | 4.680 | <.001 | -2.900 | .002 | .469 | -.402 | 1.341 | Equivalent |
| Diastolic blood pressure | Indoor vs Urban | MET | ±1.5 mmHg | -4.423  (212) | <.001 | -1.451 | .074 | -7.395 | 1.000 | -2.232 | -3.066 | -1.399 | Different from zero, but does not exceed SESOI |
| Diastolic blood pressure | Indoor vs Nature | MET | ±1.5 mmHg | -3.546  (212) | <.001 | -.861 | .195 | -6.231 | 1.000 | -1.981 | -2.904 | -1.058 | Different from zero, but does not exceed SESOI |
| Diastolic blood pressure | Urban vs Nature | EQU | ±1.5 mmHg | .485  (212) | .628 | 3.381 | <.001 | -2.411 | .008 | .251 | -.605 | 1.107 | Equivalent |
| Heart rate | Indoor vs Urban | EQU | ±2.0 bpm | .971  (212) | .332 | 3.457 | <.001 | -1.514 | .066 | .782 | -.548 | 2.111 | Inconclusive |
| Heart rate | Indoor vs Nature | MET | ±2.0 bpm | 3.414  (212) | <.001 | 5.862 | 1.000 | .965 | .168 | 2.789 | 1.439 | 4.138 | Different from zero, but does not exceed SESOI |
| Heart rate | Urban vs Nature | MET | ±2.0 bpm | 2.947  (212) | .004 | 5.884 | 1.000 | .010 | .496 | 2.007 | .882 | 3.132 | Different from zero, but does not exceed SESOI |

**Note.** PANAS = Positive and Negative Affect Schedule; POMS = Profile of Mood States; ZIPERS = Zuckerman Inventory of Personal Reactions; STICSA = State-Trait Inventory for Cognitive and Somatic Anxiety; TOST = two one-sided tests; NHST = null-hypothesis significance testing; EQU = equivalence test; MET = minimum-effect test; SESOI = smallest effect size of interest. Paired TOST procedures were used to operationalize equivalence and minimum-effect testing for the three within-subject setting contrasts. Mean differences were calculated as the second setting minus the first setting in each contrast. Raw bounds were defined separately by outcome family as the largest differences considered practically negligible in the original metric of each measure. For equivalence tests, statistical equivalence was concluded only when both one-sided TOST p values were significant. For minimum-effect tests, conclusions were based on the one-sided test corresponding to the expected direction of the contrast; the opposite one-sided test is reported for completeness but was not used to determine the conclusion. Coding key: Equivalent = both one-sided TOST p values were significant and the conventional NHST was non-significant; Equivalent, but different from zero = both one-sided TOST p values were significant and the conventional NHST was significant; Different, not equivalent = the conventional NHST was significant, but equivalence was not supported; Exceeds SESOI = the observed difference exceeded the prespecified smallest effect size of interest in the expected direction; Different from zero, but does not exceed SESOI = the conventional NHST was significant, but the minimum-effect test was not; Inconclusive = neither equivalence nor a minimum relevant effect was established.
